# Effects of Nurse-Led Multifactorial Care to Prevent Disability in Community-Living Older People: Cluster Randomized Trial

**DOI:** 10.1371/journal.pone.0158714

**Published:** 2016-07-26

**Authors:** Jacqueline J. Suijker, Marjon van Rijn, Bianca M. Buurman, Gerben ter Riet, Eric P. Moll van Charante, Sophia E. de Rooij

**Affiliations:** 1 Department of General Practice, Academic Medical Center, Amsterdam, The Netherlands; 2 Department of Internal Medicine, section of Geriatric Medicine, Academic Medical Center, Amsterdam, The Netherlands; 3 University Center for Geriatric Medicine, University Medical Center Groningen, Groningen, The Netherlands; Vanderbilt University, UNITED STATES

## Abstract

**Background:**

To evaluate the effects of nurse-led multifactorial care to prevent disability in community-living older people.

**Methods:**

In a cluster randomized trail, 11 practices (n = 1,209 participants) were randomized to the intervention group, and 13 practices (n = 1,074 participants) were randomized to the control group. Participants aged ≥ 70 years were at increased risk of functional decline based on a score ≥ 2 points on the Identification of Seniors at Risk- Primary Care, ISAR-PC. Participants in the intervention group received a systematic comprehensive geriatric assessment, and individually tailored multifactorial interventions coordinated by a trained community-care registered nurse (CCRN) with multiple follow-up home visits. The primary outcome was the participant’s disability as measured by the modified Katz activities of daily living (ADL) index score (range 0–15) at one year follow-up. Secondary outcomes were health-related quality of life, hospitalization, and mortality.

**Results:**

At baseline, the median age was 82.7 years (IQR 77.0–87.1), the median modified Katz-ADL index score was 2 (IQR 1–5) points in the intervention group and 3 (IQR 1–5) points in the control group. The follow-up rate was 76.8% (n = 1753) after one year and was similar in both trial groups. The adjusted intervention effect on disability was -0.07 (95% confidence interval -0.22 to 0.07; p = 0.33). No intervention effects were found for the secondary outcomes.

**Conclusions:**

We found no evidence that a one-year individualized multifactorial intervention program with nurse-led care coordination was better than the current primary care in community-living older people at increased risk of functional decline in The Netherlands.

**Trial Registration:**

Netherlands Trial Register NTR2653

## Introduction

The need to prevent disability and functional decline in later life is increasingly urgent with the ageing of society, the increase of multimorbidity and growing strain on limited resources.[1] Disability is defined as difficulty of or dependence in (instrumental) daily activities essential for independent living.[2] The occurrence of new physical disabilities is often called functional decline.[3] Older individuals consider prevention of disability as a patient-relevant outcome.[4] Progressive disability is associated with loss of quality of life,[5] loss of independence,[6] and high healthcare utilization.[7]

It has been suggested that a proactive, integrated care provision for community-dwelling older people is needed to address complex care needs, enable independent living and improve quality of life.[8–10] Earlier meta-analyses and reviews demonstrated that interventions including a comprehensive geriatric assessment (CGA), multifactorial interventions, and multiple follow-up visits had beneficial effects on overall functioning, especially for the relatively young, pre-frail subjects.[11–14] Nevertheless, more recent primary care studies on complex elderly care showed neutral findings.[15–21] Despite controversies over the effectiveness of multifactorial interventions to prevent functional decline,[11–13, 22–24] different proactive strategies are already part of national policies in several Western countries, including the United Kingdom and Denmark.[25]

In 2008, the Dutch government launched the National Care for the Elderly Programme (NCEP) stimulating innovative healthcare projects focused on older people with multifactorial care needs to promote physical, mental and social health and wellbeing.[26] Designing an intervention to prevent or postpone disability and functional decline, we aimed to target those who were likely to benefit most; a younger pre-frail population.[2, 11, 12, 27] Furthermore, to enhance the benefit of the target population, we identified those at increased risk of functional decline, and combined this with interventions based on current evidence or guidelines, patient-centered care, and nurse-led care coordination.[2, 11, 12, 27, 28] In a cluster randomized trial, which is part of the NCEP, we studied the effects of a systematic CGA, and nurse-led care coordination of individualized multifactorial interventions with multiple follow-up visits on preventing disability in community-living older people at increased risk of functional decline.

## Materials and Methods

The protocol for this study[28] and CONSORT checklist are available as supporting information (S1 File and S2 File). We provide a summary of the materials and methods in the current article because a more extensive description is published in the study protocol.[28]

### Design and setting

Between December 2010 and May 2014 we conducted a cluster randomized trial with a 1-year intervention and a 2-year follow-up in the north-west of the Netherlands. We invited 95 general practices who had not implemented nurse-led care coordination for community-living older people to participate. Twenty-four general practices were willingly to participate and were randomized. Community-care registered nurses (CCRN) provided the multifactorial intervention program to participants from practices allocated to the intervention group. The control group received no extra care or information besides usual care. The trial was registered at Trial Registration NTR2653. (http://www.trialregister.nl)

### Participants

All participating general practitioners (GP) selected their patients aged 70 years and over from their electronic medical record and excluded those who had a life expectancy of less than three months, suffered from dementia, did not understand Dutch, planned to move or spend a long time abroad, or lived in a nursing home. The selected persons received a self-reporting questionnaire, including a screening instrument: Identification of Seniors At Risk—Primary Care (ISAR-PC) (S1 Text).[29] ISAR-PC was developed to identify community-living older persons at increased risk of functional decline. It comprises three dichotomous items (age, dependence in instrumental activities of daily living (IADL), and impaired memory). ISAR-PC discriminates moderately and is well calibrated (Area under the receiver operating characteristics curve (AUC) range 0.63–0.64 in an independent validation cohort; p-value for calibration range 0.09–0.78; 34% of those screened were identified at increased risk (score ≥ 2). ISAR-PC was validated in Dutch. All eligible participants signed a written informed consent before inclusion. The study has been approved by the Medical Ethics Committee of the Academic Medical Center, University of Amsterdam, The Netherlands (protocol ID MEC10/182).

### Randomization and blinding

An independent statistician performed the computerized cluster randomization, stratified on the basis of socio-economic status, number of participants and general practices in both study groups.[28] Participants were blinded for the study intervention by applying a postponed informed consent procedure to prevent selection bias.[30] All outcome assessors were blinded to treatment allocation and were not otherwise involved in the study.

### Intervention

The participants in the intervention group received a systematically administered CGA, an individually tailored care treatment plan (CTP) consisting of multifactorial interventions, and nurse-led care coordination with multiple follow-up visits. The CGA, CTP and follow-up visits were conducted by the same CCRN. In total, 15 experienced CCRNs, employed by one homecare organization, participated in the intervention. All CCRNs followed a formal 10-day training in providing integrated elderly care in the community, prior to the start of the study (more information of the training is provided in S2 Text).

The CCRN conducted the CGA during a home visit. The CGA focused on somatic, psychological, functional and social domains, representing conditions such as urinary incontinence, memory problems, fall risk, and loneliness. The physical examination and performance tests of the CGA included measurement of body mass index, blood pressure and pulse (all geriatric conditions are described in S3 Text).[28] The participants were asked whether they recognized the identified conditions as relevant problems, whether they desired (additional) care or treatment for them, and in case of multiple problems, which one(s) should have priority in the CTP. To create uniformity, further diagnostic assessments and interventions came from a toolkit containing standardized evidence-based protocols and were developed by a multidisciplinary expert panel (examples of possible diagnostics and interventions are described in S3 Text).[28] Possible interventions were referral to a GP, referral to a paramedic, giving advice, follow-up visit by the CCRN. Subsequently, the CCRN discussed the yield of the CGA with the GP, and a CTP was created in which all actions expected of the participant, CCRN and/or GP were specified. The CCRN evaluated the CTP during several follow-up visits.[28]

Nurse-led care coordination consisted of elements of case management, self-management and patient-centered care, which were derived from several chronic care models.[4, 28, 31, 32] During the intervention, the CCRN worked in close collaboration with the GP and maintained contact with other healthcare professionals (e.g., occupational therapists, physiotherapists, etc.) and the participant’s caregiver(s).

To meet the demands and needs of older persons, in accordance with NCEP study guidelines, a panel of elderly people was actively involved in the design and evaluation of the study.[26]

### Care as usual

The participants from general practices randomized to the control group received usual care (S4 Text). Throughout the study, we monitored all participants’ resources utilization (Table 1 Baseline variables of participants).

**Table 1:** Distribution of baseline variables of participants with an ISAR-PC score ≥ 2, by study arm (N = 2283).

Characteristics		Intervention group	Control group
		N = 1209	N = 1074
		n(%)	n(%)
Age, in years, median (IQR)		82.6 (76.8–86.8)	82.9 (77.3–87.3)
Female sex		789 (65.2)	671 (62.7)
Caucasian		1141 (95.4)	1022 (96.5)
Level of education			
	Primary school or less	255 (21.3)	281 (26.6)
	Secondary education	760 (63.7)	648 (61.4)
	College or university	179 (15.0)	127 (12.0)
Socio-economic status			
	Low (≤1SD)	57 (4.7)	78 (7.3)
	Intermediate	931 (76.9)	890 (83.2)
	High (≥1SD)	223 (18.4)	102 (9.5)
Married/living together		561 (46.7)	489 (46.0)
Living situation			
	Independent, alone	530 (44.0)	467 (43.9)
	Independent, together	535 (44.5)	442 (41.6)
	Home for elderly	138 (11.5)	154 (14.5)
Multimorbidity (≥2)		997 (83.2)	856 (80.6)
Polypharmacy (≥3)		830 (69.3)	748 (70.7)
Modified Katz-ADL index (range 0–15), (median (IQR))		2 (1–5)	3 (1–5)
Katz-ADL (range 0–6), median (IQR)		1 (0–1)	1 (0–1)
IADL scale (range 0–7), median (IQR)		1 (0–3)	2 (0–3)
EuroQol-5D (range -0.33–1.0), mean (SD)		0.75 (0.21)	0.72 (0.22)
Emotional wellbeing (Rand-36) (range 4–100), mean (SD)		71.4 (17.4)	70.3 (17.6)
Self-perceived quality of Life (scale range 0–10), mean (SD)		7.2 (1.3)	7.2 (1.2)
Health care utilisation in past 12 months			
	Hospital admission (≥1)	306 (26.1)	264 (25.6)
	GP after hours (≥1)	232 (20.1)	175 (17.2)
	Home care (physical)	193 (17.0)	149 (14.7)
	Home care (instrumental)	654 (56.3)	523 (51.9)
	Day care	26 (2.2)	36 (3.5)
Falls (≥1) in past 12 months		418 (34.9)	344 (32.7)
Identification of seniors at risk-primary care (range 0–7.5), median (IQR)		4 (3–5)	4 (3–5)

Values are Values are numbers (percentages) unless stated otherwise.

### Baseline data collection and measurements of outcomes

The baseline assessment included demographics, socio-economic status score, comorbidities, disability (modified Katz-ADL index score),[33] health-related quality of life (EQ-5D)[34], emotional wellbeing subscale (RAND-36),[35] self-perceived quality of life,[36] healthcare utilization (hospitalization, after-hours primary care),[36] and incidence of falls within 12 months. Socio-economic status score (SES) was based on income, employment and educational level, calculated for the postal code of the participants’ residence by the Netherlands Institute for Social Research (SCP). In both groups, participants received similar self-reporting questionnaires at baseline and at six-month intervals, for two years.

### Primary and secondary outcomes

The primary outcome was participants’ change in disability measured with the 15-item modified Katz-ADL index score at one year follow-up.[33] This index is a combination of six basic ADL items based on the Katz-ADL index (bathing, dressing, toileting, transfer, incontinence and eating), seven instrumental ADL (IADL) items based on the Lawton Scale (housekeeping, meal preparation, shopping, telephone use, transportation, medication use, budgeting), and two additional items (grooming and walking). Scores range from zero to 15 points with higher scores indicating more dependence.[33] We determined the (mean) smallest meaningful change to be -0.5 points on the modified Katz-ADL index score based on previous research.[12]

The secondary outcomes were the participants’ change in health-related quality of life (EQ-5D), emotional wellbeing subscale (RAND-36), self-perceived quality of life, healthcare utilization, number of falls at all follow-up moments, and all-cause mortality. The EQ5D is a five-dimension scale to estimate preference-based health-related quality of life values. Possible health states were converted in a utility score, using a Dutch general population validation study.[37] Self-perceived quality of life was assessed using a Cantril’s Ladder where respondents rated their present quality of life on a scale between zero and ten.[36] All outcome measures were validated for the Dutch population.[36]

### Adherence to the study protocol

The intervention group’s adherence to the protocol was based on 1) the percentage of participants that received both the CGA and their personalized care and treatment plan; 2) the percentage of the CGAs that the CCRN discussed with the GP; and 3) the percentage of participants that received an evaluation of the CTP after one year.

### Sample size calculation

The sample size was based on observational data from primary care practices from a prospective cohort study (mean modified Katz-ADL score 2.70, SD 2.55),[29] which would represent an effect size of 0.20. With an assumed intracluster coefficient (ICC) of 0.015 and an expected cluster size of 100 participants per practice, the design effect was estimated at 2.50 (1+100*0.015). Using a two-sided alpha of 0.05 and power of 80%, 1,025 participants were needed in each group. The final target sample of participants was increased to 1,281 per treatment group to allow for a dropout rate of 20% within one year.[28]

### Statistical analyses

Analyses were performed according to the intention-to-treat principle. Baseline characteristics of participants were described for the two study groups. Mixed linear and negative binomial regression models with a random intercept for participants were used for continuous (modified Katz-ADL index score, EQ-5D, Rand 36, self-perceived quality of life) and count data (number of hospital admissions, after-hours primary care contacts, and falls), respectively. An additional random intercept at the GP level did not improve model fit (likelihood-ratio test p = 0.20). Linear mixed regression models employed robust standard errors to account for skewness in the outcome variable.[38] The models were adjusted for confounding variables, which were selected on the basis of causal diagrams for the various outcomes. All adjustment variables concerned baseline values of (i) the outcome variable, (ii) age, (iii) sex, (iv) (three levels of) education, and (v) (three levels of) socio-economic status.[39] Based on the likelihood-ratio test (p_interaction_ < 0.05) interaction terms for treatment × time were added, to assess whether the treatment effect, if any, varied over time. Kaplan-Meier curves were used to estimate survival rates and compared using the log-rank test.

Post-hoc analyses were performed by adding interaction terms for treatment × levels of education (high and intermediate), treatment × socio-economic status (low and intermediate), treatment × levels of age (75–79 years, 80–84 years, 85–89 years, and 90 years and over), and treatment × baseline disability (tertiles of the Katz score) to the fully adjusted model (p_interaction_ < 0.05). In the treatment group, we visually explored the mean change on the modified Katz-ADL index between baseline and one year as a function of the number of home visits, the number of interventions, and the variation among the 15 community care nurses in the intervention group.

To assess the impact of missing data, we performed a sensitivity analysis. We created ten imputation sets by multiple imputation using chained equations and predictive mean matching (PMM) in STATA 13.[40] Missing values were imputed separately for each study group. We then repeated the fully adjusted mixed regression models on the ten sets and combined the estimates using Rubin’s rule. We used IBM SPSS, Version 20.0 (IBM Corp. 2011) and STATA 13 (StataCorp. 2013. College Station, TX) for data analysis.

## Results

**Fig 1:**
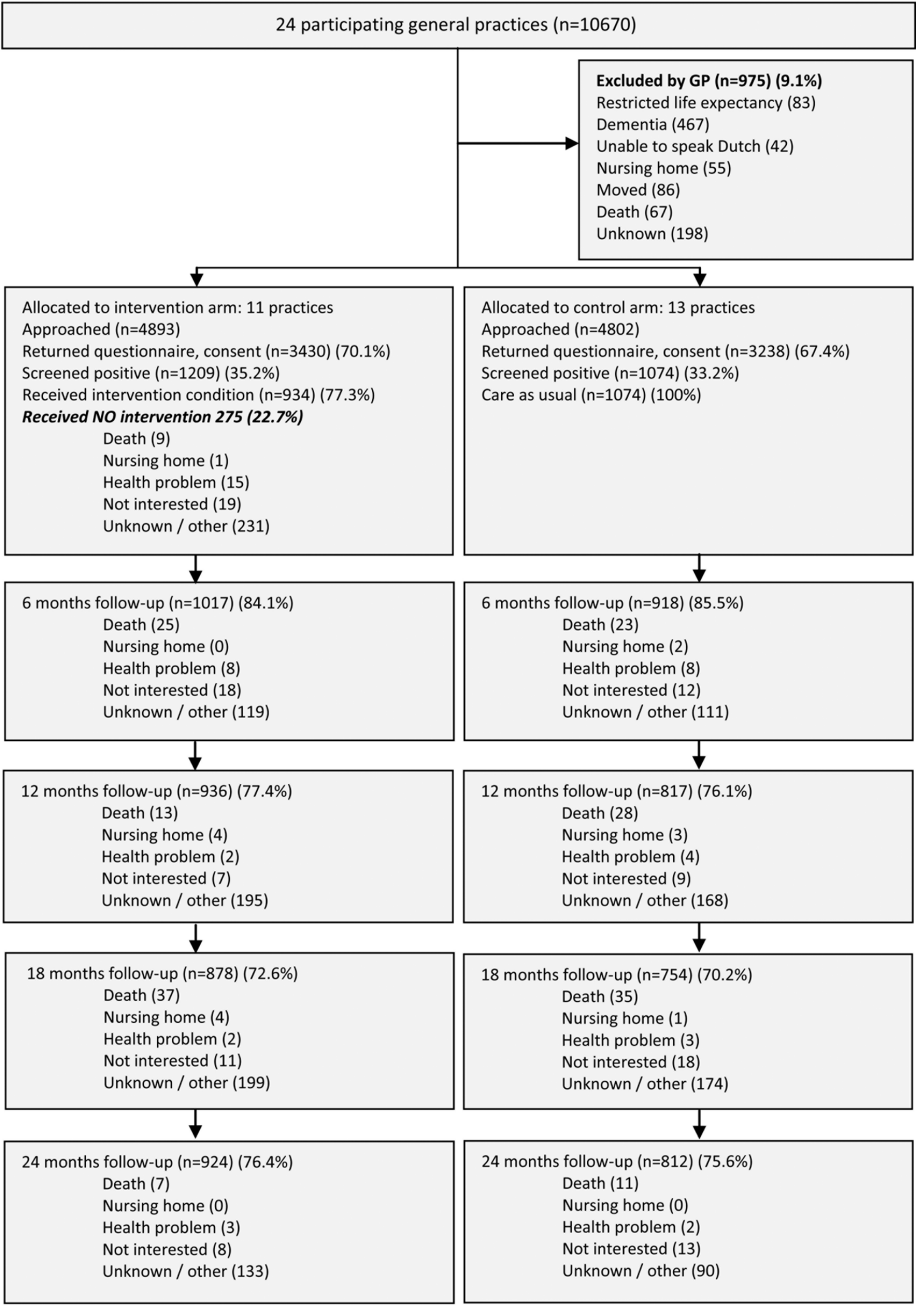
Flow of practices and participants through the trial. Numbers do not add up because persons who did not return a questionnaire at 6 months (n = 119) could return a questionnaire at 12, 18 and/or 24 months. Eleven practices were randomized to the intervention group and 13 practices were randomized to the control group. In both groups around 35% of the invited persons were at increased risk of functional decline and participated in the study. In both groups the follow-up rates were around 77% and 76% after one and two years respectively.

Eleven practices were randomized to the intervention group and 13 practices were randomized to the control group. Screening with ISAR-PC resulted in 35.2% (1209/3430) of the participants in the intervention group and 33.2% (1074/3238) of the participants in the control group (Fig 1 Flow chart). The follow-up rates after one year were 77.4% (936/1209) in the intervention group and 76.1% (817/1074) in the control group (Fig 1 Flow chart). The characteristics of the participants and general practices are shown in Table 1 Baseline variables of participants, and S1 Table and S2 Table. The participants’ baseline characteristics were balanced between both study groups except that the intervention group showed a higher percentage of people with a high SES level (Table 1). The median age of the participants was almost 83 years in both groups. The median modified Katz-ADL index score was 2 (IQR 1–5) points in the intervention group and 3 (IQR 1–5) points in the control group. The participants who declined the comprehensive geriatric assessment (n = 275) were older, had more (I)ADL disabilities, and more often lived in a home for the elderly (S3 Table).

The prevalence of geriatric conditions identified by the CGA is shown in S4 Table. The mean number of problems identified in the CGA was 6.4 (SD 2.8). The median number of geriatric conditions that were recognized as a problem was 1 (IQR 0–2). Geriatric conditions that were mostly recognized as a problem were pain, depressive symptoms, hearing impairment and loneliness. The median number initiation of interventions was 1 (IQR 0–2). Most interventions were initiated for pain, incontinence, mobility, fall risk and loneliness. Reasons for no intervention are shown in S5 Table.

Eleven practices were randomized to the intervention group and 13 practices were randomized to the control group. In both groups around 35% of the invited persons were at increased risk of functional decline and participated in the study. In both groups the follow-up rates were around 77% and 76% after one and two years respectively.

### Adherence to the protocol

Among all participants, older people 77.0% (934/1209) received a CGA and 76.6% (926/1209) received a CTP (Fig 1 Flow chart). The CCRN discussed 61.6% (575/934) of the CGAs with the general practitioner. The CGAs that were not discussed with the GP (38.4%; 359/934) involved participants declining care or with no unmet care needs. After one year, 77.4% (698/898) of the CTPs were evaluated with the participants (S5 Table). During the intervention, the mean number of home visits was 3.2 (SD 1.5). A more detailed description of the adherence to the protocol will be described in the process evaluation of the study and will be published separately.

### Primary outcome

**Fig 2:**
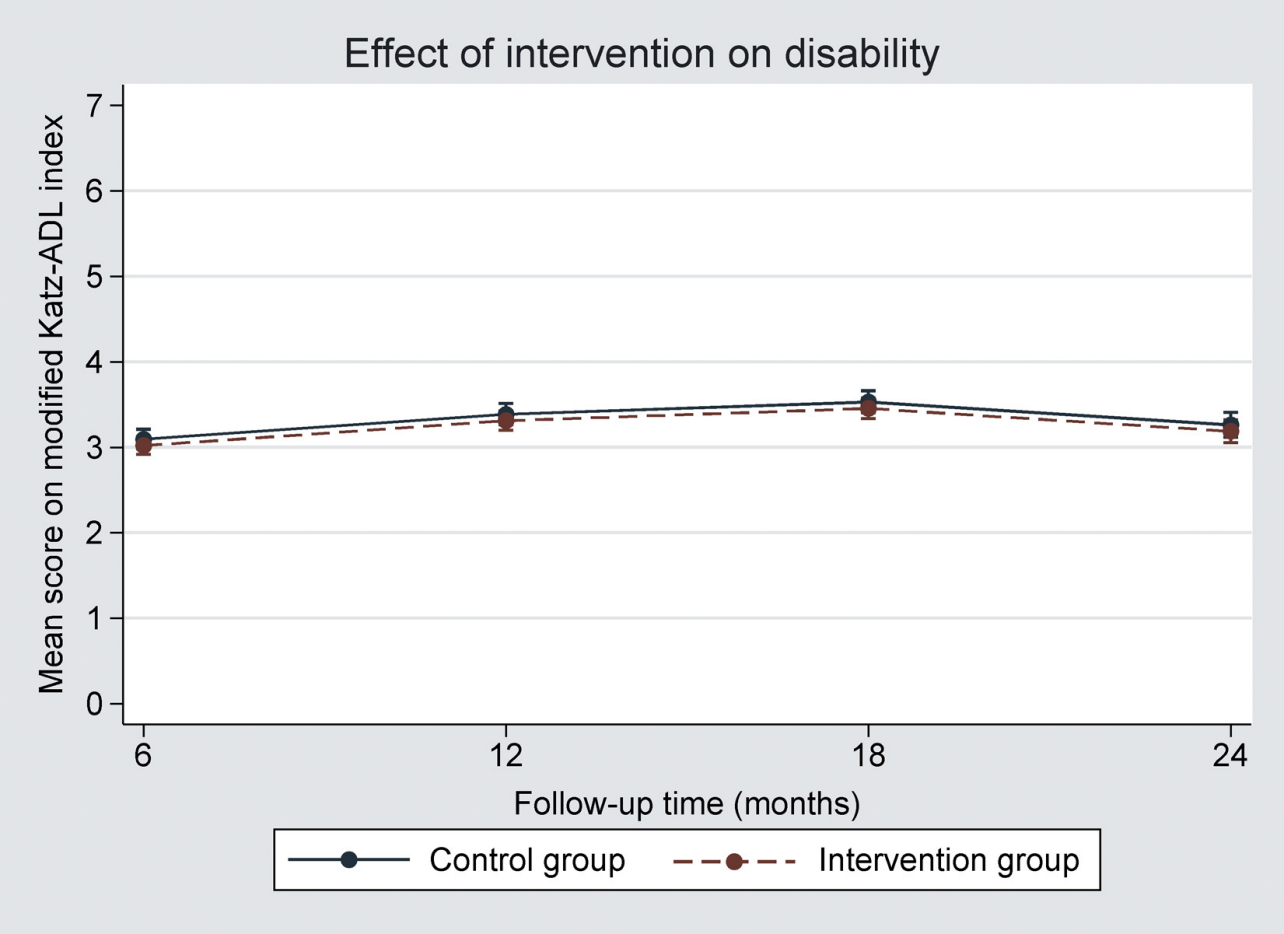
Effect of the intervention on disability (modified Katz-ADL index score). The effect of the intervention was equal (-0.07) and very small across all follow-up times; The scale on the y-axis only ranges from 0 to 7.0 points on the modified Katz-ADL index score, which is a 15-point scale.

One year after the start, the mean modified Katz-ADL index score had increased in both groups, indicating an increase in disability. The effect of the intervention after one year, adjusted for baseline modified Katz-ADL score, age, sex, SES and level of education, was -0.07 (95% confidence interval (CI) -0.22 to 0.07) (Table 2 Primary results of trial, Fig 2 Effect of the intervention on disability). The results of the unadjusted and adjusted analyses are presented in S6 Table. The sensitivity analyses accounting for missing data gave similar results (S7 Table).

**Table 2:** Primary results of trial: Mean scores and difference between intervention and control arm at 6, 12, 18 and 24 months.

Outcome	Baseline		6 months		12 months		18 months		24 months		6, 12, 18, 24 months		
	Mean score		Mean score		Mean score		Mean score		Mean score		Mean difference	p-value	ICC (SE)
	(95% CI)		(95% CI)		(95% CI)		(95% CI)		(95% CI)		(95% CI)		
	Intervention	Control	Intervention	Control	Intervention	Control	Intervention	Control	Intervention	Control	Time × intervention (p_interaction_ = 0.68)		
Modified Katz-ADL index (0–15)	3.13	3.34	3.02	3.09(	3.31)	3.39	3.46	3.53	3.19	3.27	-0.07	0.33	0.47 (0.01)*
	(2.97–3.29)	(3.15–3.52)	(2.92–3.12)	(2.98–3.21)	(3.20–3.42)	(3.26–3.51)	(3.33–3.58)	(3.40–3.66)	(3.05–3.32)	(3.12–3.41)	(-0.22–0.07)		

Estimated mean scores and mean difference between intervention and control arm adjusted for baseline variables, which were selected on the basis of causal diagrams. Analysis was adjusted for age, sex, socio-economic status, level of education, and modified Katz-ADL index score.

ICC = intracluster coefficient participant level.

* based on random-intercept (participants) model. On a two-level model (general practice, participant) the ICC was 0.003.

### Secondary outcomes

**Fig 3:**
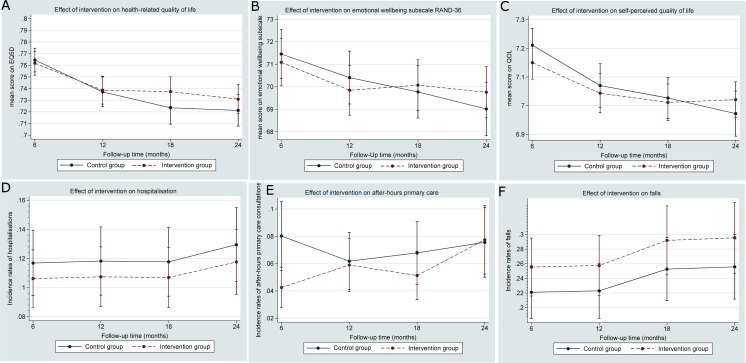
Effect of the intervention on secondary outcomes. The effect of the intervention was small and statistically not significant for all secondary outcomes across all follow-up moments except for after-hours primary care were a small effect was found at six months. Note that the scales on the y-axis do not cover the full range of the measurement instrument.

**Fig 4:**
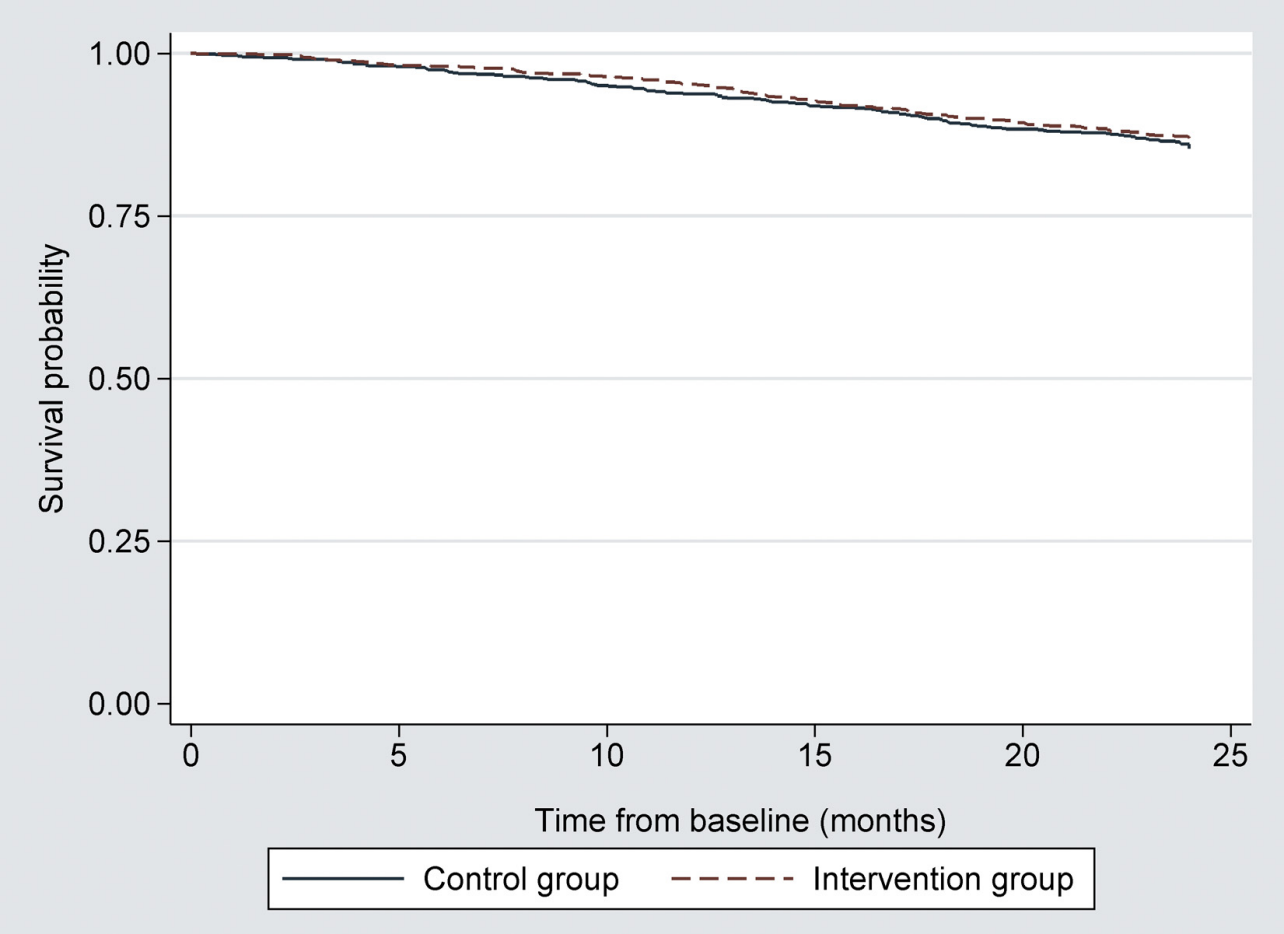
Kaplan Meier all-cause mortality for persons at increased risk of functional decline. No difference was found in overall mortality between both study arms; Log rank test = 0.84; p = 0.36.

At each follow-up moment, we found neither clinically nor statistically significant intervention effects for health-related quality of life (EQ5D), emotional wellbeing, self-perceived quality of life, or number of hospitalizations or falls (Fig 3 Effect of intervention on secondary outcome and S8 Table, S9 Table and S10 Table). At six months, the incidence rate ratio for after-hours use of primary care was 0.53 (95% CI 0.36 to 0.77). This effect had disappeared at one year. Unadjusted and adjusted analyses for the secondary outcomes are presented in S8 Table, S9 Table and S10 Table. We found no intervention effect on all-cause mortality (Fig 4 Kaplan Meier all cause mortality).

### Post-hoc analyses

No interactions were found between treatment group and level of education, level of socio-economic status, baseline level of disability or baseline level of age in the post-hoc analyses (S11 Table). Furthermore, we explored the variation between CCRNs (all in the intervention group) with regard to the primary outcome. Overall, the mean changes in disability score were similar across the 13 CCRNs. We also explored the existence of “dose-response” effects by the number of home visits and interventions in the CTP but found none (S1 Fig, S2 Fig and S3 Fig).

## Discussion

In this cluster randomized trial, we found no evidence that a one-year individualized multifactorial intervention program with nurse-led care coordination was better than current primary care in community-living older people at increased risk of functional decline in The Netherlands. Additionally, the intervention was not more effective than current primary care for all other outcomes assessed.

### Strengths and limitations of this study

A major strength of the study is that, given that we avoided major bias, it robustly excluded clinically relevant effects of the intervention on the primary outcome. Specifically, the 95% confidence interval around the mean difference between the two treatment groups (-0.07; 95% CI, −0.22 to 0.07) excluded the predefined functional decline of -0.5 points by a wide margin. Thus, although the study was not designed as a non-inferiority trial, we found evidence of no effect. The sensitivity analyses accounting for missing data confirmed the robustness of the main analyses. To prevent bias in the outcome assessment, outcome assessors and participants were blinded using a postponed informed consent procedure. Another strength is the patient-centered approach comprising recognition and prioritization of identified problems for care and treatment. Addressing problems that older persons consider important may increase adherence to the intervention and facilitate implementation. Other strengths of the study include the active involvement of older people in the design and evaluation of the study, the high participation rate, the high adherence rate to the structured study protocol, and the evidence-based toolkit.

The study also has some limitations. First, the CCRNs and the GPs could not be blinded for the purpose of the study because they were part of the intervention. Second, despite computerized randomization the study showed some imbalance in baseline disability and SES. To overcome this, we adjusted the analyses for SES and baseline value of the outcome measure. Third, in the intervention group 23% of the participants declined to take part in the CGA. Although we collected reasons for non-participation/declining (Fig 1 Flow chart), a large number did not fill in the reason for decline (n = 150 unknown), or could not be contacted (n = 81 other reasons). Overall, the participants who declined CGA were older, had more (I)ADL disabilities, and more often lived in a home for the elderly compared to the participants who received the CGA (S3 Table). These non-respondents may have caused underestimation of the overall effect, as analyses were intention to treat. Fourth, not all parts of the intervention were implemented as planned. According to the nurses’ registration, not all CGAs were discussed with the GPs. These CGAs involved participants declining care or without unmet care needs. This may have caused underestimation of the adherence to the intervention, and thus of the overall effect. A detailed more qualitative process evaluation is therefore needed to gain more insight in the motivation and morale towards adherence to the protocol.

### Comparison with other studies

Two recent meta-analyses on multifactorial interventions,[12, 41] and one meta-analysis on preventive home visits[24] have demonstrated small effects on functional decline. However, these results should be interpreted with caution due to heterogeneity in the target population, the large variability of possible interventions and the variation in outcome measurements of ADL and IADL.[12, 24, 41] One meta-analysis demonstrated that studies that were conducted before the year 1993 showed increased risk reduction on physical function.[12] This implicates that health care systems probably improved since then, adapting principles of effective elderly care in usual care.[12] Studies performed in the United States also demonstrated increased risk reduction on functional decline, because primary care for older people is less developed in the US compared to most European countries.[41]

Recent studies in the United Kingdom, Canada, and The Netherlands found neutral effects of multifactorial preventive interventions to prevent disability or functional decline,[15–18, 20, 21] except for one study who described a small effect of nurse-led personalized care on postponing functional decline among highly educated participants.[42] The window of opportunity for multifactorial interventions to prevent functional decline may therefore be larger in countries without a well-developed primary health care, such as the US.[43]

### Explanation of the finding and implications for future research

There are several possible explanations why we did not find an effect of a one-year nurse-led multifactorial intervention. First, the intervention lasted one year and measured its potential effects over a two-year period, which may have been too short to see effects emerge. Targeting a pre-frail population, which focuses on the prevention of future incidents, such as disability or mortality, a longer intervention and follow-up period has demonstrated beneficial effects.[44–47] Furthermore, although experienced CCRNs were trained before and during the intervention, we observed that nurses needed time to build a steady collaboration with the GPs with focus on the new way of working with the GP and to focus on geriatric conditions. A detailed process evaluation is needed to learn how both professionals interacted together and which components of the intervention were or were not regarded useful to implement.

Second, insufficient contrast between the study groups could explain the lack of effect that was observed. The majority of the participants in the control group contacted their GP on a regular basis and received home care nursing (S12 Table). The Netherlands has a very easy assessable health care system,[48] and the quality of regular GP care is considered to be of high standard; evidence-based guidelines for the management of chronic conditions managed in the GP practice are available and the adherence to these protocols is good.[49] Problem-based, goal-oriented approaches might already have been incorporated in usual care and additional improvement seems difficult.[43]

Third, although the modified Katz-ADL index is able to validly and reliably measure unfavourable health outcomes,[50] more insight is needed in its ability to detect clinical relevant change in disability over time. Besides, the separate components of the interventions were developed to provide treatment and care for geriatric conditions, such as pain, incontinence, hypertension, and loneliness, but this may not have been sufficiently associated with daily functioning as such. To enhance the effect of the intervention, more emphasis should be put on interventions that can directly postpone new disabilities, such as physical activity.[44, 51] Using other measures with a closer relation to the individual outcome, such as goal-attainment scaling (GAS) might suit a patient-centered approach better.[52] GAS, is a clinimetric tool that describes goal achievement for individual patients. GAS has demonstrated to detect clinically important change in the evaluation of complex interventions in frail elderly patients.[52]

Fourth, the intensity of the intervention may have been too low to see effects emerge. However, we found no “dose-response” effects by the number of home visits and interventions in the CTP.

Fifth, although many geriatric conditions were identified in the CGA, only one condition per participant was recognized as a problem and only one intervention was initiated. This could indicate that the CGA detected many conditions without unmet needs. Older persons may simply accept certain conditions as a normal part of ageing, or perhaps they were already addressed. Furthermore, prioritizing geriatric conditions may have resulted in a selection of interventions, while unfavorable conditions where left untreated. However, recognition and prioritizing may be useful in developing a person-centred approach to care, potentially facilitating shared decision-making and overall efficiency.

Finally, although we found a small and transient effect of the intervention for after-hours GP care use, we think that this should be interpreted with caution because of the relatively large number of outcome measures assessed.

## Conclusions

In this cluster randomized trial, we found no evidence that a one-year multifactorial nurse-led care program was better than current primary care in community-living older people at increased risk of functional decline in The Netherlands. Nevertheless, the implementation of preventive programs in general practice is ongoing in many health care systems throughout the Western world. We may learn from process evaluations why Dutch general practitioners want to implement preventive interventions despite the apparent ineffectiveness of a one-year intervention on functional decline over and above current primary care. In consideration of the ageing of Western societies, increasing task delegation from GPs to nurses warrants further non-inferiority analyses on both quality and costs, and warrants evaluation from a societal perspective to explore whether such programs may still deliver valuable services at acceptable costs and efforts.

## Supporting Information

S1 Fig Mean changes in modified Katz-ADL index scores among nurses. (DOC)

S2 Fig Mean changes in modified Katz-ADL index scores by increasing number of interventions performed. (DOC)

S3 Fig Mean changes in modified Katz-ADL index scores by increasing number of home visits. (DOC)

S1 File Study protocol. (DOC)

S2 File CONSORT checklist. (DOCX)

S1 Table Characteristics of participants and general practices in the intervention group. (DOC)

S2 Table Characteristics of participants and general practices in the control group. (DOC)

S3 Table Characteristics of participants in the intervention group who received or declined the comprehensive geriatric assessment. (DOC)

S4 Table Prevalence of geriatric conditions in CGA. (DOC)

S5 Table Adherence to the trial protocol. (DOC)

S6 Table Primary results of trial: Mean scores and difference between intervention and control arm at 6, 12, 18 and 24 months. (DOC)

S7 Table Primary results of trial: Mean difference between intervention and control group at 12 months after accounting for missing values. (DOC)

S8 Table Mean scores and differences between intervention and control group at 6, 12, 18 and 24 months for secondary outcomes health related quality of life and emotional wellbeing. (DOC)

S9 Table Incidence rates and rate ratio’s for intervention and control group at 6, 12, 18 and 24 months for secondary outcomes hospitalization and falls. (DOC)

S10 Table Incidence rates and rate ratio’s for intervention and control group at 6, 12, 18 and 24 months for secondary outcome after-hours primary care. (DOC)

S11 Table Interaction terms of different levels of education, socio-economic status, baseline disability, and age. (DOC)

S12 Table In hours general practice care during follow-up for older persons at increased risk of functional decline (ISAR-PC≥2). (DOC)

S1 Text Identification of Senior At Risk- Primary Care (ISAR-PC) screening instrument. (DOC)

S2 Text Training of nurses participating in the trial. (DOC)

S3 Text Evidence-based protocols used in the trial. (DOC)

S4 Text Care as usual in the Dutch healthcare system. (DOC)

## References

[1] Cesari M, Vellas B, Hsu FC, Newman AB, Doss H, King AC, et al A Physical Activity Intervention to Treat the Frailty Syndrome in Older Persons-Results From the LIFE-P Study. The journals of gerontology Series A, Biological sciences and medical sciences. 2014 Epub 2014/11/13. 10.1093/gerona/glu099 . 25387728 PMC4311184

[2] Fried LP, Ferrucci L, Darer J, Williamson JD, Anderson G. Untangling the concepts of disability, frailty, and comorbidity: implications for improved targeting and care. The journals of gerontology Series A, Biological sciences and medical sciences. 2004;59(3):255–63. Epub 2004/03/20. . 15031310 10.1093/gerona/59.3.m255

[3] Beaton K, McEvoy C, Grimmer K. Identifying indicators of early functional decline in community-dwelling older people: A review. Geriatrics & gerontology international. 2014 Epub 2014/10/11. 10.1111/ggi.12379 . 25303103

[4] Fried TR, Tinetti ME, Iannone L, O'Leary JR, Towle V, Van Ness PH. Health outcome prioritization as a tool for decision making among older persons with multiple chronic conditions. Archives of internal medicine. 2011;171(20):1854–6. 10.1001/archinternmed.2011.424 . 21949032 PMC4036681

[5] Groessl EJ, Kaplan RM, Rejeski WJ, Katula JA, King AC, Frierson G, et al Health-related quality of life in older adults at risk for disability. American journal of preventive medicine. 2007;33(3):214–8. 10.1016/j.amepre.2007.04.031 17826582 PMC1995005

[6] Hardy SE, Allore HG, Guo Z, Dubin JA, Gill TM. The effect of prior disability history on subsequent functional transitions. JGerontolA BiolSciMedSci. 2006;61(3):272–7. 61/3/272 [pii]. 10.1093/gerona/61.3.272 16567377

[7] Fried TR, Bradley EH, Williams CS, Tinetti ME. Functional disability and health care expenditures for older persons. Archives of internal medicine. 2001;161(21):2602–7. . 11718592 10.1001/archinte.161.21.2602

[8] Multimorbidity AGSEPotCoOAw. Guiding principles for the care of older adults with multimorbidity: an approach for clinicians: American Geriatrics Society Expert Panel on the Care of Older Adults with Multimorbidity. Journal of the American Geriatrics Society. 2012;60(10):E1–E25. 10.1111/j.1532-5415.2012.04188.x 22994865 PMC4450364

[9] Netherlands HCot. Health care for the elderly with multimorbidity. 2008.

[10] Rechel B, Grundy E, Robine JM, Cylus J, Mackenbach JP, Knai C, et al Ageing in the European Union. Lancet. 2013;381(9874):1312–22. 10.1016/S0140-6736(12)62087-X . 23541057

[11] Stuck AE, Egger M, Hammer A, Minder CE, Beck JC. Home visits to prevent nursing home admission and functional decline in elderly people: systematic review and meta-regression analysis. JAMA: the journal of the American Medical Association. 2002;287(8):1022–8. jma10044 [pii]. 11866651 10.1001/jama.287.8.1022

[12] Beswick AD, Rees K, Dieppe P, Ayis S, Gooberman-Hill R, Horwood J, et al Complex interventions to improve physical function and maintain independent living in elderly people: a systematic review and meta-analysis. Lancet. 2008;371(9614):725–35. S0140-6736(08)60342-6 [pii]; 10.1016/S0140-6736(08)60342-6 18313501 PMC2262920

[13] Huss A, Stuck AE, Rubenstein LZ, Egger M, Clough-Gorr KM. Multidimensional preventive home visit programs for community-dwelling older adults: a systematic review and meta-analysis of randomized controlled trials. The journals of gerontology Series A, Biological sciences and medical sciences. 2008;63(3):298–307. . 18375879 10.1093/gerona/63.3.298

[14] Liebel DV, Friedman B, Watson NM, Powers BA. Review of nurse home visiting interventions for community-dwelling older persons with existing disability. Medical care research and review: MCRR. 2009;66(2):119–46. Epub 2008/12/31. 10.1177/1077558708328815 . 19114607

[15] Blom J, den Elzen W, van Houwelingen AH, Heijmans M, Stijnen T, Van den Hout W, et al Effectiveness and cost-effectiveness of a proactive, goal-oriented, integrated care model in general practice for older people. A cluster randomised controlled trial: Integrated Systematic Care for older People-the ISCOPE study. Age Ageing. 2016;45(1):30–41. 10.1093/ageing/afv174 26764392 PMC4711660

[16] Hoogendijk EO, van der Horst HE, van de Ven PM, Twisk JW, Deeg DJ, Frijters DH, et al Effectiveness of a Geriatric Care Model for frail older adults in primary care: Results from a stepped wedge cluster randomized trial. Eur J Intern Med. 2015 10.1016/j.ejim.2015.10.023 . 26597341

[17] Looman WM, Fabbricotti IN, de Kuyper R, Huijsman R. The effects of a pro-active integrated care intervention for frail community-dwelling older people: a quasi-experimental study with the GP-practice as single entry point. BMC geriatrics. 2016;16(1):43 Epub 2016/02/18. 10.1186/s12877-016-0214-5 ; PubMed Central PMCID: PMCPmc4755064. 26879893 PMC4755064

[18] Metzelthin SF, van Rossum E, de Witte LP, Ambergen AW, Hobma SO, Sipers W, et al Effectiveness of interdisciplinary primary care approach to reduce disability in community dwelling frail older people: cluster randomised controlled trial. Bmj. 2013;347:f5264 Epub 2013/09/12. 10.1136/bmj.f5264 ; PubMed Central PMCID: PMCPmc3769159. 24022033 PMC3769159

[19] van Hout HP, Jansen AP, van Marwijk HW, Pronk M, Frijters DF, Nijpels G. Prevention of adverse health trajectories in a vulnerable elderly population through nurse home visits: a randomized controlled trial [ISRCTN05358495]. The journals of gerontology Series A, Biological sciences and medical sciences. 2010;65(7):734–42. 10.1093/gerona/glq037 . 20457579

[20] Fletcher AE, Price GM, Ng ES, Stirling SL, Bulpitt CJ, Breeze E, et al Population-based multidimensional assessment of older people in UK general practice: a cluster-randomised factorial trial. Lancet. 2004;364(9446):1667–77. S0140-6736(04)17353-4 [pii]; 10.1016/S0140-6736(04)17353-4 15530627

[21] Ploeg J, Brazil K, Hutchison B, Kaczorowski J, Dalby DM, Goldsmith CH, et al Effect of preventive primary care outreach on health related quality of life among older adults at risk of functional decline: randomised controlled trial. Bmj. 2010;340:c1480 Epub 2010/04/20. 10.1136/bmj.c1480 ; PubMed Central PMCID: PMCPmc3191725. 20400483 PMC3191725

[22] Bouman A, van Rossum E, Nelemans P, Kempen GI, Knipschild P. Effects of intensive home visiting programs for older people with poor health status: a systematic review. BMC health services research. 2008;8:74 10.1186/1472-6963-8-74 18387184 PMC2364620

[23] Elkan R, Kendrick D, Dewey M, Hewitt M, Robinson J, Blair M, et al Effectiveness of home based support for older people: systematic review and meta-analysis. Bmj. 2001;323(7315):719–25. 11576978 10.1136/bmj.323.7315.719 PMC56889

[24] Mayo-Wilson E, Grant S, Burton J, Parsons A, Underhill K, Montgomery P. Preventive home visits for mortality, morbidity, and institutionalization in older adults: a systematic review and meta-analysis. PloS one. 2014;9(3):e89257 10.1371/journal.pone.0089257 24622676 PMC3951196

[25] National service framework for older people London: Department of Health 2001.

[26] The National Care for the Elderly Programme Available from: http://www.nationaalprogrammaouderenzorg.nl/english/the-national-care-for-the-elderly-programme/.

[27] Ferrucci L, Guralnik JM, Studenski S, Fried LP, Cutler GB Jr., Walston JD. Designing randomized, controlled trials aimed at preventing or delaying functional decline and disability in frail, older persons: a consensus report. JAmGeriatrSoc. 2004;52(4):625–34. 10.1111/j.1532-5415.2004.52174.x [doi];JGS52174 [pii]. 15066083

[28] Suijker JJ, Buurman BM, ter Riet G, van Rijn M, de Haan RJ, de Rooij SE, et al Comprehensive geriatric assessment, multifactorial interventions and nurse-led care coordination to prevent functional decline in community-dwelling older persons: protocol of a cluster randomized trial. BMCHealth ServRes. 2012;12:85 1472-6963-12-85 [pii]; 10.1186/1472-6963-12-85 PMC3374886 22462516

[29] Suijker JJ, Buurman BM, van Rijn M, van Dalen MT, ter Riet G, van Geloven N, et al A simple validated questionnaire predicted functional decline in community-dwelling older persons: prospective cohort studies. Journal of clinical epidemiology. 2014;67(10):1121–30. 10.1016/j.jclinepi.2014.05.014 . 25103817

[30] Boter H, van Delden JJ, de Haan RJ, Rinkel GJ. Modified informed consent procedure: consent to postponed information. Bmj. 2003;327(7409):284–5. 10.1136/bmj.327.7409.284 12896945 PMC1126664

[31] Berry-Millett R, Bodenheimer TS. Care management of patients with complex health care needs. Synth Proj Res Synth Rep. 2009;(19). 52372. doi: 52372 22052205

[32] Bodenheimer T, Lorig K, Holman H, Grumbach K. Patient self-management of chronic disease in primary care. JAMA: the journal of the American Medical Association. 2002;288(19):2469–75. . 12435261 10.1001/jama.288.19.2469

[33] Weinberger M, Samsa GP, Schmader K, Greenberg SM, Carr DB, Wildman DS. Comparing proxy and patients' perceptions of patients' functional status: results from an outpatient geriatric clinic. JAmGeriatrSoc. 1992;40(6):585–8. 10.1111/j.1532-5415.1992.tb02107.x 1587975

[34] EuroQol G. EuroQol—a new facility for the measurement of health-related quality of life. Health policy. 1990;16(3):199–208. . 10109801 10.1016/0168-8510(90)90421-9

[35] Aaronson NK, Muller M, Cohen PD, Essink-Bot ML, Fekkes M, Sanderman R, et al Translation, validation, and norming of the Dutch language version of the SF-36 Health Survey in community and chronic disease populations. Journal of clinical epidemiology. 1998;51(11):1055–68. . 9817123 10.1016/s0895-4356(98)00097-3

[36] Lutomski JE, Baars MA, Schalk BW, Boter H, Buurman BM, den Elzen WP, et al The development of the Older Persons and Informal Caregivers Survey Minimum DataSet (TOPICS-MDS): a large-scale data sharing initiative. PloS one. 2013;8(12):e81673 10.1371/journal.pone.0081673 24324716 PMC3852259

[37] Lamers LM, Stalmeier PF, McDonnell J, Krabbe PF, van Busschbach JJ. [Measuring the quality of life in economic evaluations: the Dutch EQ-5D tariff]. Nederlands tijdschrift voor geneeskunde. 2005;149(28):1574–8. . 16038162

[38] Maas CJHJJ. The influence of violations of assumptions on multilevel parameter estimates and their standard errors. Computational statistics and data analysis. 2004;46:427–40.

[39] Williamson EJ, Aitken Z, Lawrie J, Dharmage SC, Burgess JA, Forbes AB. Introduction to causal diagrams for confounder selection. Respirology (Carlton, Vic). 2014;19(3):303–11. Epub 2014/01/23. 10.1111/resp.12238 . 24447391

[40] White IR, Royston P, Wood AM. Multiple imputation using chained equations: Issues and guidance for practice. Stat Med. 2011;30(4):377–99. 10.1002/sim.4067 . 21225900

[41] Lin JS, Whitlock EP, Eckstrom E, Fu R, Perdue LA, Beil TL, et al Challenges in synthesizing and interpreting the evidence from a systematic review of multifactorial interventions to prevent functional decline in older adults. Journal of the American Geriatrics Society. 2012;60(11):2157–66. 10.1111/j.1532-5415.2012.04214.x . 23072282

[42] Blijenberg N. Personalized Primary Care for Older Persons: An evaluation of a multicomponent nurse-led care program Utrecht: University of Utrecht; 2013.

[43] Iliffe S. Community-based interventions for older people with complex needs: time to think again? Age Ageing. 2016;45(1):2–3. 10.1093/ageing/afv185 . 26764386

[44] Pahor M, Guralnik JM, Ambrosius WT, Blair S, Bonds DE, Church TS, et al Effect of structured physical activity on prevention of major mobility disability in older adults: the LIFE study randomized clinical trial. JAMA: the journal of the American Medical Association. 2014;311(23):2387–96. Epub 2014/05/29. 10.1001/jama.2014.5616 ; PubMed Central PMCID: PMCPmc4266388. 24866862 PMC4266388

[45] Lindholm E, Brevinge H, Haglind E. Survival benefit in a randomized clinical trial of faecal occult blood screening for colorectal cancer. The British journal of surgery. 2008;95(8):1029–36. 10.1002/bjs.6136 . 18563785

[46] Stuck AE, Moser A, Morf U, Wirz U, Wyser J, Gillmann G, et al Effect of health risk assessment and counselling on health behaviour and survival in older people: a pragmatic randomised trial. PLoS medicine. 2015;12(10):e1001889 10.1371/journal.pmed.1001889 26479077 PMC4610679

[47] Thompson SG, Ashton HA, Gao L, Buxton MJ, Scott RA. Final follow-up of the Multicentre Aneurysm Screening Study (MASS) randomized trial of abdominal aortic aneurysm screening. The British journal of surgery. 2012;99(12):1649–56. Epub 2012/10/05. 10.1002/bjs.8897 ; PubMed Central PMCID: PMCPmc3569614. 23034729 PMC3569614

[48] Netherlands tops European healthcare index. Bmj. 2016;352(i538):BMJ 2016;352:i538. 10.1136/bmj.i538 26818884

[49] Grol R. Successes and failures in the implementation of evidence-based guidelines for clinical practice. Medical care. 2001;39(8 Suppl 2):Ii46–54. Epub 2001/10/05. . 11583121 10.1097/00005650-200108002-00003

[50] Laan W, Zuithoff NP, Drubbel I, Bleijenberg N, Numans ME, de Wit NJ, et al Validity and reliability of the katz-15 scale to measure unfavorable health outcomes in community-dwelling older people. The journal of nutrition, health & aging. 2014;18(9):848–54. Epub 2014/11/13. 10.1007/s12603-014-0479-3 . 25389963 PMC12878471

[51] Tak E, Kuiper R, Chorus A, Hopman-Rock M. Prevention of onset and progression of basic ADL disability by physical activity in community dwelling older adults: a meta-analysis. Ageing research reviews. 2013;12(1):329–38. Epub 2012/10/16. 10.1016/j.arr.2012.10.001 . 23063488

[52] Rockwood K, Howlett S, Stadnyk K, Carver D, Powell C, Stolee P. Responsiveness of goal attainment scaling in a randomized controlled trial of comprehensive geriatric assessment. Journal of clinical epidemiology. 2003;56(8):736–43. Epub 2003/09/05. . 12954465 10.1016/s0895-4356(03)00132-x

