# Supplementary material for: Effects of Nurse-Led Multifactorial Care to Prevent Disability in Community-Living Older People: Cluster Randomized Trial
Source: PLoS One. 2016 Jul 26;11(7):e0158714. doi: 10.1371/journal.pone.0158714 (PMC4961429; doi:10.1371/journal.pone.0158714)
Supplement: S1 Fig — (DOC) [file pone.0158714.s001.doc]

**S1 Fig. Mean changes in modified Katz-ADL index scores at 12 months between nurses**

Adjusted for age, sex, socio-economic status, level of education, baseline score on modified Katz-ADL index and multimorbidity.
The vertical line represents the mean change in the modified Katz-ADL index score for all nurses.
