# Supplementary material for: Effects of Nurse-Led Multifactorial Care to Prevent Disability in Community-Living Older People: Cluster Randomized Trial
Source: PLoS One. 2016 Jul 26;11(7):e0158714. doi: 10.1371/journal.pone.0158714 (PMC4961429; doi:10.1371/journal.pone.0158714)
Supplement: S1 File — (DOC) [file pone.0158714.s004.doc]

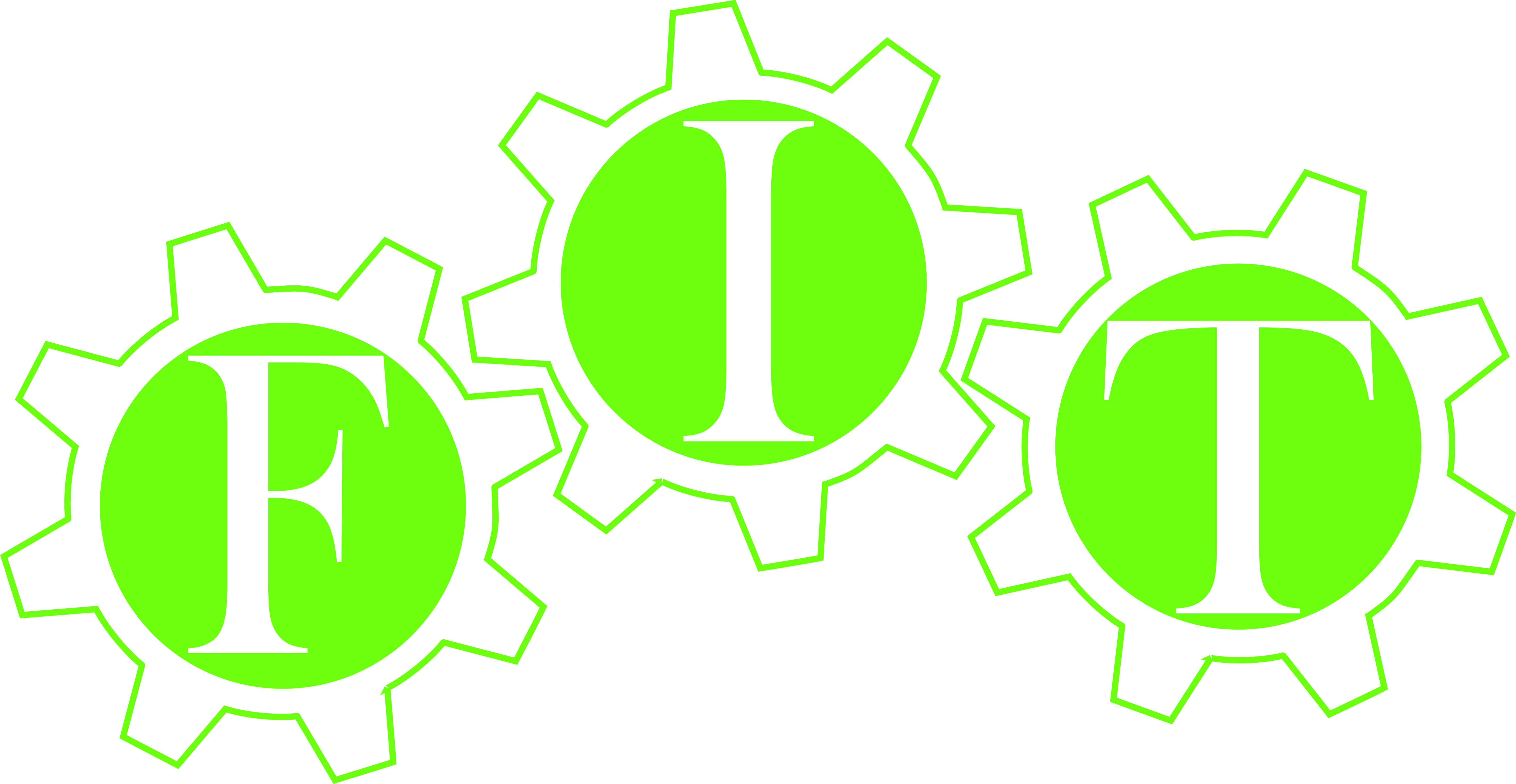


# S1 FILE STUDY PROTOCOL

**Functional decline In Transition (FIT):**

**A cluster randomised trial to prevent disability in community-dwelling older people.**

# PROTOCOL TITLE “FIT-study”

| **Protocol ID** | **Functional decline In Transition** |
| --- | --- |
| **Short title** | **FIT** |
| **Version** | **1** |
| **Date** | **August 16, 2010** |
| **Coordinating investigator/project leader** | **Sophia E. de Rooij/ Eric Moll van Charante** |
| **Principal investigator(s) (in Dutch: hoofdonderzoeker/uitvoerder)** | **AMC: Sophia E. de Rooij en Eric Moll van Charante** |
|  |  |
| **Sponsor (in Dutch: verrichter/opdrachtgever)** | **ZonMW, national program of care for the older patients, grant no: 60-61900-98-270** |
|  |  |
| **Independent physician(s)** | **N. van der Velde** |
|  |  |
|  |  |
| **Laboratory sites** | **Not applicable** |
|  |  |
|  |  |
| **Pharmacy** | **Not applicable** |
|  |  |

## PROTOCOL SIGNATURE SHEET

| **Name** | **Signature** | **Date** |
| --- | --- | --- |
| **For non-commercial research,**  **Head of Department:** | **Prof. dr. E. Schadé, head of the department of General Practice** |  |
| **Coordinating Investigator/Project leader/Principal Investigator:** | **Dr. Sophia E. de Rooij, principal investigator, geriatrician** |  |
|  |  |  |

## TABLE OF CONTENTS

Functional decline In Transition (FIT) 1

1. PROTOCOL TITLE ‘FIT’ [2](#__RefHeading___Toc257376711)

1.1 PROTOCOL SIGNATURE SHEET [3](#__RefHeading___Toc257376712)

1.2 TABLE OF CONTENTS [4](#__RefHeading___Toc257376713)

1.3 LIST OF ABBREVIATIONS AND RELEVANT DEFINITIONS [6](#__RefHeading___Toc257376714)

1.4 SUMMARY [8](#__RefHeading___Toc257376715)

2. INTRODUCTION AND RATIONALE [10](#__RefHeading___Toc257376716)

3. OBJECTIVES 11

4. STUDY POPULATION 12

4.1 Population (base) and setting 12

4.2 Inclusion criteria 12

4.3 Exclusion criteria 12

4.4 Sample size calculation 12

5. STUDY DESIGN 13

6. TREATMENT OF SUBJECTS 14

7. METHODS 17

7.1 Study parameters/endpoints 17

7.1.1 Main study parameter/endpoint 17

7.1.2 Secondary study parameters/endpoints 17

7.1.3 Other study parameters 17

7.2 Randomisation 19

7.3 Study procedures 20

7.4 Withdrawal of individual subjects 21

7.5 Replacement of individual subjects after withdrawal 21

7.6 Follow-up of subjects withdrawn from treatment 21

7.7 Premature termination of the study 21

8. SAFETY REPORTING 22

8.1 Section 10 WMO event 22

8.2 Adverse and serious adverse events 22

8.3 Follow-up of adverse events 23

8.4 Data Safety Monitoring Board (DSMB) 23

9. STATISTICAL ANALYSIS 24

10. ETHICAL CONSIDERATIONS 25

10.1 Regulation statement 25

10.2 Recruitment and consent 25

10.3 Benefits and risks assessment, group relatedness 25

10.4 Compensation for injury 25

10.5 Incentives 25

11. ADMINISTRATIVE ASPECTS AND PUBLICATION 26

11.1 Handling and storage of data and documents 26

11.2 Amendments 26

11.3 Annual progress report 26

11.4 End of study report 26

11.5 Public disclosure and publication policy 27

12. REFERENCES 28

APPENDIX 1 Diagnostic Comprehensive Geriatric Assessment (summary)...........................................32

APPENDIX 2 Postponed informed consent procedure …………………………………………………………….……....33

## LIST OF ABBREVIATIONS AND RELEVANT DEFINITIONS

| **ABR** | **ABR form, General Assessment and Registration form, is the application form that is required for submission to the accredited Ethics Committee (In Dutch, ABR = Algemene Beoordeling en Registratie)** |
| --- | --- |
| **ACOVE** | **Assessing Care Of Vulnerable elderly people** |
| **ADL** | **Activities of daily living** |
| **AE** | **Adverse Event** |
| **ALDS** | **AMC Linear Disability Score** |
| **AR** | **Adverse Reaction** |
| **CA** | **Competent Authority** |
| **CCMO** | **Central Committee on Research Involving Human Subjects; in Dutch: Centrale Commissie Mensgebonden Onderzoek** |
| **CGA** | **Comprehensive Geriatric Assessment** |
| **CN** | **Community Care Nurse** |
| **CRU** | **Clinical Research Unit** |
| **CV** | **Curriculum Vitae** |
| **DSMB** | **Data Safety Monitoring Board** |
| **EU** | **European Union** |
| **EudraCT** | **European drug regulatory affairs Clinical Trials** |
| **GCP** | **Good Clinical Practice** |
| **GP** | **General practitioner** |
| **HCN** | **Health Care Nurse (specialized in elderly care in general practice)** |
| **IADL** | **Instrumental Activities of Daily Living** |
| **IB** | **Investigator’s Brochure** |
| **IC** | **Informed Consent** |
| **ICOVE** | **Improving Care of Vulnerable elderly people** |
| **IMP** | **Investigational Medicinal Product** |
| **IMPD** | **Investigational Medicinal Product Dossier** |
| **ISAR-PC** | **Identification of Seniors at Risk in Primary Health Care** |
| **KOZ** | **Kring ouderenzorg** |
| **METC** | **Medical research ethics committee (MREC); in Dutch: medisch ethische toetsing commissie (METC)** |
| **NHP** | **Nursing Home Physician** |
| **NS** | **Clinical Nurse Specialist** |
| **OLVG** | **Onze Lieve Vrouwe Gasthuis** |
| **(S)AE** | **(Serious) Adverse Event** |
| **SPC** | **Summary of Product Characteristics (in Dutch: officiële productinfomatie IB1-tekst)** |
| **Sponsor** | **The sponsor is the party that commissions the organisation or performance of the research, for example a pharmaceutical**  **company, academic hospital, scientific organisation or investigator. A party that provides funding for a study but does not commission it is not regarded as the sponsor, but referred to as a subsidising party.** |
| **SUSAR** | **Suspected Unexpected Serious Adverse Reaction** |
| **Wbp** | **Personal Data Protection Act (in Dutch: Wet Bescherming Persoonsgevens)** |
| **WMO** | **Medical Research Involving Human Subjects Act (in Dutch: Wet Medisch-wetenschappelijk Onderzoek met Mensen** |

## SUMMARY

**Rationale:** Maintenance of functioning is an important feature of healthy aging. However, decline in functioning is common in community-dwelling older people. This can be related to an acute event, such as a hospital admission, or a more chronic condition, such as chronic disease, such as arthrosis. Most older people have capabilities to recover, but once certain (instrumental) activities of daily living (I)ADL appear to be lost, older people are at increased risk for further decline. New evidence shows that complex interventions can help elderly people to independently continue living at home, largely through prevention of the need for nursing-home care.

**Objective:** To prevent functional decline in community-dwelling elderly people of 70 years and older, through (1) screening for increased risk for functional decline; followed by

(2) a nurse-led comprehensive geriatric assessment (CGA) in patients at increased risk for functional decline, a tailor-made care and treatment plan, and seven follow-up contacts during one year.

**Study design:** A multicenter, cluster randomized clinical trial at the level of the General Practitioner (GP) comparing a pro-active, multi-component (multidisciplinary and multidimensional) intervention, coordinated by a Health Care Nurse (HCN) specialized in elderly care with care as usual.

**Study population:** Community-dwelling elderly people 70 years and older with an increased risk for functional decline.

**Intervention**: First, all eligible elderly people who are registered with their GP will be sent a postal questionnaire, the Identification of Seniors at Risk in Primary Care (ISAR-PC), that was developed during a pilot study. In half of the GP practices, patients with increased risk for functional decline will be invited to receive a nurse-led comprehensive geriatric assessment (CGA). In the CGA, participants will be screened for over 30 conditions on four domains (physical, functional, mental, social functioning) that are most prevalent among elderly people. The targeted problems are part of an evidence based protocol (‘toolkit’) that was developed in the Defence-study ([www.defencestudy.nl](http://www.defencestudy.nl/)) and further extended in a pilot phase of the FIT-study, and yields a care and treatment plan that is discussed with both patient and GP. When consensus is reached on the intervention, the HCN will coordinate all care and treatment contacts and will frequently see all participating elderly in the office or at home to monitor the effects of all interventions.

**Main study parameters/endpoints:** The main outcome is the the level of (instrumental) activities of daily living, measured with the modified Katz ADL index score. Secondary outcomes include hospital and nursing home admissions, self-reported health care utilization and quality of life (EQ-6D) and overall mortality.

**Nature and extent of the burden and risks associated with participation, benefit and group relatedness**

All elderly people with increased risk for functional loss will be subjected to a comprehensive geriatric assessment (appendix 2) that will approximately last one hour and is expected to take place in the GPs' surgery or at home (if the patient is not able to come to the GP practice). The identified problems are discussed with the GP and the patient and are translated into a care and treatment plan. Examples of potential interventions are consultations of an ergotherapist or physiotherapist for elderly people with increased fall-risk and/or impairments in their mobility. The nurses aim for 7 follow-up contacts within one year, through office consultations, home visits or telephone contact if warranted. The practice nurse will work in close collaboration with the GP, evaluating the care and treatment plans in the course of the follow-up, while looking out for emerging new problems over time.

Participants in the control group may not benefit from filling out the questionnaires, although their response will be analyzed over time and will be made available to their GP toward the end of the study, thus yielding potential new (or existing) problems that can be (further) addressed.

2. INTRODUCTION AND RATIONALE

In old age, reduction in physical function can lead to loss of independence, the need for hospital and long-term nursing-home care, and premature death. The importance of physical, functional, psychological, and social factors in realizing a healthy old age is recognized by elderly people, health-care professionals, and policy makers.

The risk factors for reduced physical function in elderly people, as identified in longitudinal studies, relate to comorbidities, physical and psychosocial health, environmental conditions, social circumstances, nutrition, and lifestyle. The need for a preventive strategy based around identification and treatment of diverse risk factors have been recognized in many western countries and many trials of complex interventions have been performed over the last decades. Complex interventions can be regarded as a combination of interdisciplinary teamwork for health and social problems. Trials have focused on general and frail elderly populations, elderly people discharged from hospital, and those at risk of falling. However, the development of risk factors, admission to hospital, and risk of falling represent a common chain of experiences for many elderly people. Likewise, multifactorial interventions in these populations have common characteristics and, in addition to targeting specific outcomes relating to hospital readmissions and falls, share the common aims of physical function maintenance, disability limitation, and promotion of independence.

Geriatric screening and multidimensional assessment are recognized in many European health care systems. In the US, managed care organisations focus mainly on frail elderly people and those discharged from hospital. Care is coordinated by case managers and this model has been applied in other countries, including England, where all individuals aged 75 and older receive a yearly assessment through nurse-led case management of elderly people.

Currently, many initiatives in the field of primary care for community-dwelling elderly people directed at prevention of functional loss and maintenance of independence are unfolding in the Netherlands, but whether or not health care nurses (HCN) should play a leading role in the coordination of care is still subject of debate. Nevertheless, it appears that comprehensive, integrated care for elderly people with (multiple) chronic conditions is lacking. Therefore, the regional geriatric network of the Academic Medical Centre in Amsterdam ('Kring Ouderenzorg AMC (KOZ)') has instigated the FIT-study (Functional decline In Transition). This study comprises a general screening to identify elderly people with increased risk for functional decline and a further diagnostic CGA to find problems that can be addressed through a care and treatment plan.

3. OBJECTIVES

#### Primary Objective

To prevent functional decline in community-dwelling elderly people of 70 years and older.

#### Secondary objectives

To prevent hospital admissions (1), nursing home admissions (2), disability (3), to improve quality of health and quality of life (4), and to describe met and unmet care needs (elderly and care-givers) (5).

4. STUDY POPULATION

**4.1. Population (base) and setting**

The study takes place in the Dutch primary health care setting in a region north of Amsterdam (region Alkmaar) and in some GP practices within the city of Amsterdam (North and South-East). All elderly subjects of 70 and older within the participating GP practices are invited to participate in the study. The structure of the Dutch healthcare system, in which virtually all inhabitants are registered with a GP, minimizes selection bias at this stage. The only exclusion criteria are prevalent dementia and disorders or circumstances expected to

interfere with successful participation in the geriatric assessments and follow-up.

Within the participating general practices a Health Care Nurse (HCN) who has specific and broad expertise in elderly care will play a central role in the assessments, development of a care and treatment plan and coordination of the provided care for the patient with all health care professionals involved.

**4.2 Inclusion criteria**

Phase 1 (general screening):

- Patients aged 70 years and older

Phase 2 (intervention)

- An increased risk for functional decline, defined as a score of two or more on the ISAR-PC screening instrument
- Speaks and understands Dutch
- Patient is registered with a GP

**4.3 Exclusion criteria**

- Terminal illness
- Dementia
- Does not speak or understand Dutch
- Living in a nursing home

**4.4 Sample size calculation**

The smallest difference with meaningful clinical relevance was determined at 0.5 points on the Katz-ADL15 (primary endpoint). Based on data from the pilot, this would represent a small effect size only (Cohen’s effect size of 0.20).

In this study, the intervention takes place at the level of the general practice, so this is also the unit of randomisation (cluster). Using a two-sided alpha of 0.01 and power of 90% (to keep the chances of spurious associations from multiple comparisons to a minimum) and assuming a design-effect of 1.08, in total 850 elderly people would need to receive both CGA and follow-up care. To allow for a drop-out rate of 40% (including patients who do not want treatment for identified problems, and patients who withdraw or die in the course of the study), in total around 1418 elderly people will have to receive CGA at the start of the intervention.

5.0 STUDY DESIGN

**5.1 Study design**

A multicenter, cluster randomized clinical trial at the level of the General Practitioner (GP) comparing a pro-active, multi-component (multidisciplinary and multidimensional) intervention, coordinated by a Health Care Nurse (HCN) with care as usual.

##### Figure 1 flow chart of patient selection and randomisation (next page)

##### 5.2 Duration of the study and follow-up

##### The study will start September 1, 2010. The screening phase will take 6 months and the following intervention 12 months (including all intended 7 follow-up contacts). After 12 months, all elderly people in both arms of the trial will receive a postal questionnaire to score the modified Katz ADL index. To assess long-term effects, a similar postal questionnaire will be send after 18 and 24 months. The final analysis will be reported around September 2013.

**Total population ≥70 yrs**

**n=8220**

**Baseline measurement**

**n=5670**

**Increased risk: ±50%**

**Invitation for CGA**

**n=1418 (100%)**

**CGA**

**n=1134 (80%)**

**Intervention: 10/11 GP practices; N≈2835**

**Exclusion (6%, n=658):**

- Restricted life expectancy

- Dementia

- Planning to move

- Unable to speak Dutch

**Non respondents**

**(25%, n=1891)**

Declining to participate or unable to contact

**Follow-up measurements**

**Katz-ADL/ALDS**

**Hospital/nursing home admissions**

**Quality of Life**

(6, 12, 18, 24 months)

**Eligible elderly people receiving ISAR-PC**

**n=7562**

**Control: 10/11 GP practices;**

**N≈2835**

**Non- respondents**

**(20%, n=284/1418)**

**No care/tr. plan**

**(25%, n=284/1134)**

**Care/treatment**

**7 follow-up contacts**

**n=850 (60%)**

**Intervention:**

**CGA and care/treatment starting with ±50% of total population in intervention practices**

6. TREATMENT OF SUBJECTS

I SCREENING OF THE ELDERLY POPULATION

First, all eligible elderly people who are registered with their GP will be sent a postal questionnaire, the Identification of Seniors at Risk in Primary Care (ISAR-PC), containing 4 yes/no questions. This instrument originally consisted of 6 yes/no questions, i.e. on problems with activities of daily life, memory, or recent falls. It was developed to predict the risk for functional loss of patients attending the Accident and Emergency Room (McCusker, 1999) and modified for the primary care setting during a pilot phase of the current study.

II INTERVENTION: THE CARE COORDINATION PROGRAMME

The core of the intervention (health care coordination) concerns the activities of the health care nurse (HCN), that has been specifically designed with/for elderly people. Integrating components from disease management and case management, the HCN offers an evidence based care and treatment plan, tailored to each individual patient.

The primary care geriatric team (HCN and the general practitioner (GP)) will bear active responsibility for implementing the health care coordination. The practice nurse is an experienced, registered nurse (5^th^ expertise level) with a special focus on elderly care.

The health care coordination programme consists of two phases:

1) Postal screening for an increased risk of functional decline (ISAR-PC).

2a) An increased ISAR-PC score (≥2) is followd by a comprehensive geriatric assessment (CGA) focussing on over 30 geriatric problems that are most prevalent in primary health care (measured in 4 domains: physical, functional, mental, and social functioning).

2b) After the CGA an individual care and treatment plan aims for the prevention of (further) functional decline. For the systematic identification of geriatric problems and proposition of subsequent interventions and integrated protocol is used, referred to as ‘the toolkit’. It was developed during the Defence-study (www.defencestudy.nl) and further extended and modified during the pilot phase of this study (see also Appendix 2 and 3). The toolkit contains internationally validated instruments to identify geriatric problems (such as the Mini Mental State Examination and the Geriatric Depression Scale; see Appendix 2 for overview). The interventions proposed in the toolkit are evidence-based or based on current best practices. The intervention will be worked out in detail and implemented for each problem field. The provision of information, support, advice and/or instructions may be related to medication adherence, fall prevention, etc. but also to the consultation of other health care professionals. For example, in case of poly-pharmacy, the pharmacist may be involved; when the fall-risk appears to be increased an ergotherapist will be asked to scrutinize the home for accident-prone elements, and in case of loneliness or lack of meaningful activities, contact with a social worker can be instigated to explore the opportunities for improved social participation. A consultation with a nursing home physician or a visit to the geriatric outpatient clinic can also be part of this process. The care and treatment plan will always be talked through with the patient (and/or informal caregiver if appropriate) first to explore his/her willingness to participate in any form of improvement plan, after which advice from the GP will be obtained.

7. METHODS

**7.1 Study parameters/endpoints**

The primary outcome measure is the level of (instrumental) activities of daily living (baseline-12 months), measured with the modified Katz ADL index (15 items).

### 7.1.1 Main study parameter/endpoint

See 7.1

### 7.1.2 Secondary study parameters/endpoints

Secondary outcomes will be differences in hospital admissions, nursing home admissions and disability (measured with the AMC Linear Disability Scale, ALDS) after 12 months. Likewise, differences in quality of life will be studied (EQ-5D, RAND 36) as well as met and unmet care needs (CANE) (see also ‘minimal data set’: [www.nationaal](http://www.nationaal/) programmaouderenzorg.nl/toolkit) [NB The ALDS is a generic and validated continuous scale that has scores ranging from 0 to 100, with a lower score expressing more impairments in daily functioning.]

### 7.1.3 Other study parameters

The presence of geriatric conditions that are assessed will be registered as co-variates in the prediction model (see appendix 2 for the exact content)

Process evaluation

Apart from the primary and secondary outcomes, supplementary qualitative and semi-qualitative data will be collected to provide insight at the professional and the network level into the practicability and feasibility of the intervention. Qualitative data will also be analysed in search of factors that could enable or inhibit the future implementation of the care programme. Further specifications follow below.

1] Professional/network level

(a) The effects of the experiment on the feasibility and continuity of the requested and offered care, on professional expertise, on experienced work pressure, on satisfaction about the organisation and on the content of the primary and secondary care that is provided. In addition, the frequency will be registered of specific requests resulting in consultation of the geriatrics adviser, the nurses working in home care, the paramedics working at the primary care level, and services at the secondary and tertiary care levels.

(b) Content of the intervention: a process evaluation will be conducted into the rate of fully implemented intervention plans. In addition, the four distinct components (physical, functional , mental, and social) of the intervention and follow-up will be evaluated separately.

2] Factors facilitating or inhibiting the implementation of the care programme

Examples of questions that will be addressed here are:

- Are all elderly from the target group being screened for their risk profile?
- Are the geriatric problems of all elderly with a positive screening (i.e. an increased risk of functional decline) being described according to the agreed system?
- Do the health care nurses (HCN) visit the elderly person at home or are participants seen in the GP office? What are the constraining factors in either routes? Is there a perceived added benefit to home visits?
- Does the HCN contact the caregivers/family?

- Do the contacts with HCN/(para)medical personnel take place within the indicated time frame?

- Do elderly persons feel that their care needs are addressed? Where is room for improvement?
- Is the composition of the primary care team around the patient appropriate, is a specific player missing, or are there perhaps too many players?

**7.2 Randomisation, blinding and treatment allocation**

This is an cluster randomized controlled trial in the Netherlands, comparing regular care as control condition with a nurse led, multi-component intervention directed at geriatric problems as the experimental condition. Whereas randomization takes place at GP level, interventions take place at the individual level on the basis of identified geriatric problems. A pre-randomization procedure will be followed to allow for the allocation of a practice nurse to the intervention practices. This requires a few months of preparation, during which the nurses will take part in a specialized elderly training program and logistical preparations will be made regarding necessary consultation rooms and equipments. The randomisation procedure will be website-based, using permuted blocks (maximum block size of 4) and stratified by median number of patients (1) and social status score (2) in each GP practice.

Patients in practices randomized to the control condition receive care as usual according to the current guidelines for Dutch general practice. At baseline, all patients will receive a postal questionnaire to assess their overall risk on functional loss. In the intervention practices, patients with increased risk profiles will be asked to participate in further geriatric assessments and follow-up through an integrated care and treatment plan.

In the control condition, the results of the postal questionnaires on patients with increased geriatric risk profiles will be made available to the GPs at the end of the intervention, to minimize contamination effects. Patients without increased risk in the in the intervention practices and all patients in the control practices will be blinded to the intervention through the use of a postponed informed consent procedure, described by Boter et al (2005). See also Figure 1 (5.1 ‘Study design’). This informed consent procedure is chosen to minimize Hawthorne effects among the patients who are not invited to attend the geriatric assessments and its successive follow-up. In the information letter to the patient, the purpose of the study will be explained, without specifically mentioning the intervention by the practice nurse. Targeted patients in the intervention group are further informed about the care coordination. After termination of the study, patients in both study groups will be informed about the research question and interventions that took place (*single-delayed consent design,* Schellings 2005).

The project will include the equivalent of 21 GP practices in the region of Alkmaar and will be randomised in a ratio of 1:1. Randomization is performed by the AMC Clinical Research Unit (CRU).

**7.3 Study procedures**

| **Measures** | **Intervention group** | **Control group** |
| --- | --- | --- |
| **<4 wks,** Extractions from electronic medical records (in Dutch: Huisarts Informatie Systeem, HIS) by GP of all patients aged 70 and over.  Screening for eligibility by GP (inclusion criteria). | **X** | **X** |
| **<2 wks** Pre-randomisation at the level of GP practices | **X** | **X** |
| **Start of study (Sept. 2010):**  Postal questionnaires (i.e. ISAR-PC, Katz-ADL, MDS), along with study information and postponed informed consent | **X** | **X** |
| **6 wks**  -Comprehensive Geriatric Assessment: (CGA; Appendix 2).  -Integrated care and treatment plan, discussed with both patient and GP  -Start of implementation care and treatment plan; follow-up contact with practice nurse (and other care givers if indicated) is planned | **X** |  |
| **6 wks – 12 months***  Follow-up contacts (7) by nurse to monitor the care and treatment plan | **X** |  |
| **6-12-18-24 months**  Postal questionnaires containing KATZ-(I)ADL, ALDS (T0/T12) and MDS. | **X** | **X** |

*** Total recruitment period will take around 6 months; therefore, the last GP practices included will end 6 months later than the first.**

**7.4 Withdrawal of individual subjects**

Subjects can leave the study at any time for any reason if they wish to do so without any consequences. The investigator can decide to withdraw a subject from the study for urgent medical reasons.

**7.5 Replacement of individual subjects after withdrawal**

A total of 850 patients are needed to receive CGA, individual care plan and follow-up during one year to obtain sufficient power. After correction for 40% drop-out (see Figure p.14), around 1418 participants are needed to enrol in the CGA.

**7.6 Follow-up of subjects withdrawn from treatment**

If the study subject withdraws from study participation, he or she will be receiving geriatric consultation visits as part of the usual provided patient care. Follow-up will take place by telephone.

**7.7 Premature termination of the study**

As the intervention is directed towards improving care provided to elderly people, it appears unlikely that the study will be terminated prematurely.

8.0 SAFETY REPORTING

**8.1 Section 10 WMO event**

In accordance to section 10, subsection 1, of the WMO, the investigator will inform the subjects and the reviewing accredited METC if anything occurs, on the basis of which it appears that the disadvantages of participation may be significantly greater than was foreseen in the research proposal. The study will be suspended pending further review by the accredited METC, except insofar as suspension would jeopardise the subjects’ health. The investigator will take care that all subjects are kept informed.

**8.2 Adverse and serious adverse events**

Adverse events are defined as any undesirable experience occurring to a subject during the study, whether or not considered related to the experimental treatment (For example, an adverse event can also be related to a diagnostic procedure or to an already existing condition)*.* All adverse events reported spontaneously by the subject or observed by the investiga­tor or his staff will be recorded, or reported by the Healt Care Nurse or GP. Mortality and hospital admission rates will be monitored in both conditions and reported to the METC at regular intervals or immediately if necessary.

A serious adverse event (SAE) is any untoward medical occurrence or effect that at any dose:

- results in death;
- is life threatening (at the time of the event);
- requires hospitalisation;
- results in persistent or significant disability or incapacity;
- is a congenital anomaly or birth defect;
- is a new event of the trial likely to affect the safety of the subjects, such as an unexpected outcome of an adverse reaction, lack of efficacy of an IMP used for the treatment of a life threatening disease, major safety finding from a newly completed animal study, etc.

All SAEs will be reported through the web portal *ToetsingOnline* to the accredited METC that approved the protocol, within 15 days after the sponsor has first knowledge of the serious adverse reactions.

SAEs that result in death or are life threatening should be reported expedited. The expedited reporting will occur not later than 7 days after the responsible investigator has first knowledge of the adverse reaction. This is for a preliminary report with another 8 days for completion of the report.

**8.3 Follow-up of adverse events**

All adverse events will be followed until they have abated, or until a stable situation has been reached. Depending on the event, follow up may require additional tests or medical procedures as indicated, and/or referral to the general physician or a medical specialist.

**8.4 Data Safety Monitoring Board (DSMB)**

Due to the minimal expected side effects related to the intervention a DSMB will not be instituted.

9. STATISTICAL ANALYSIS

Statistical analyses will be based on an intention-to-treat principle. Baseline assessments and outcome parameters will be summarized using simple descriptive statistics. The main analysis focuses on a comparison of patients with a similar risk profile (based on ISAR-PC and Katz-ADL scores in both groups). As the Katz-ADL data will have a correlated multilevel structure (patients are ‘nested’ within their GPs) difference between these scores will be analyzed using a linear mixed, multilevel model. If necessary, we will adjust the effect size for baseline imbalance. The same (linear and logistic) multi-level approach will be used with regard to the secondary outcome parameters, including survival rates. Survival data will be additionally analyzed using Kaplan-Meier survival curves and the log-rank test.

We will perform a predefined subgroup analysis for patients with baseline ‘high’ and ‘low’ risk scores (ISAR-PC≥2,5 and less, based on pilot-assessments) on all primary and secondary outcomes. In all analyses statistical uncertainties will be quantified via corresponding 99% confidence intervals.

Finally, process outcome data will be analyzed qualitatively within the theoretical framework of the adaptive implementation model (Meiland 2005).

10. ETHICAL CONSIDERATIONS

**10.1 Regulation statement**

The study will be conducted according to the principles of the Declaration of Helsinki

(originally adopted by the 18th WMA General Assembly, Helsinki, Finland, June 1964,

last amendment in Tokyo, 09.10.2004) and in accordance with the Medical Research Involving Human Subjects Act (WMO).

**10.2 Recruitment and consent**

Participants will be recruited from the practices of general practitioners. Eligible subjects will be notified by a letter from the study-group, with permission and signature from their GP. The investigator of the study will explain, when necessary, the rationale of the study and the study burden. An information letter and informed consent will be given to the patient and/or the primary caregiver. If no legal representative is registered for this patient, informed consent will be asked at (in the following order) the authorized representative, or the registered partner of the patient, or the adult children of the patient, or the adult brothers(s) or sister(s) of the patient. He/she will be asked to read the information carefully (see information for consent form). The (GP)practice of the patient receives a randomisation number when he/she is willing to participate or when the representative agrees on the participation of the patient. During the study, the patient can always reconsider his/her participation.

**10.3 Benefits and risks assessment, group relatedness**

*Scientific benefit*
Preventing functional decline by an effective pro-active approach may help older persons with increased risk for functional decline to maintain their independence and quality of life. Additionally, this study may offer a valuable contribution to the studies on complex, integrated intervention strategies.

**10.4 Compensation for injury**

The investigators ask for an exemption for the WMO insurance as there are no direct risks for participating in the study.

**10.5 Incentives**

Not applicable

11.0 ADMINISTRATIVE ASPECTS AND PUBLICATION

**11.1 Handling and storage of data and documents**

The data will be handled by the investigators of the study (Sophia de Rooij, Eric Moll van Charante, Jacqueline Suijker, Bianca Buurman-van Es). They will have access to the source data. One of the project leaders (SR) will have access to the randomization code. Data will be entered according to AMC Good Clinical Practice Guidelines with a number and fictional initials for each patient and a double data entry process. The communication data will be erased within six month after the termination of the study.

**11.2 Amendments**

A ‘substantial amendment’ is defined as an amendment to the terms of the METC application, or to the protocol or any other supporting documentation, that is likely to affect to a significant degree:

- the safety or physical or mental integrity of the subjects of the trial;
- the scientific value of the trial;
- the conduct or management of the trial; or
- the quality or safety of any intervention used in the trial.

All substantial amendments will be notified to the METC and to the competent authority.

Non-substantial amendments will not be notified to the accredited METC and the competent authority, but will be recorded and filed by the sponsor.

**11.3 Annual progress report**

The sponsor/investigator will submit a summary of the progress of the trial to the accredited METC once a year. Information will be provided on the date of inclusion of the first subject, numbers of subjects included and numbers of subjects that have completed the trial, serious adverse events/ serious adverse reactions, other problems, and amendments.

**11.4 End of study report**

The PI will notify the accredited METC and the competent authority of the end of the study within a period of 90 days. The end of the study is defined as the last patient’s follow up after 12 months is completed.

In case the study is ended prematurely, the PI will notify the accredited METC and the competent authority within 15 days, including the reasons for the premature termination.

Within one year after the end of the study, the PI will submit a final study report with the results of the study, including any publications/abstracts of the study, to the accredited METC and the Competent Authority.

**11.5 Public disclosure and publication policy**

## The trial will be registered in the Dutch Clinical trial registry

12.0 REFERENCES

Avila-Funes, J. A., Helmer, C., Amieva, H., Barberger-Gateau, P., Le, G. M., Ritchie, K. et al. (2008). Frailty among community-dwelling elderly people in France: the three-city study. *J.Gerontol.A Biol.Sci.Med.Sci., 63,* 1089-1096.

Avila-Funes JA, Amieva H, Barberger-Gateau P, Le Goff M, Raoux N, Ritchie K, Carrière I, Tavernier B, Tzourio C, Gutiérrez-Robledo LM, Dartigues JF.[Cognitive impairment improves the predictive validity of the phenotype of frailty for adverse health outcomes: the three-city study.](http://www.ncbi.nlm.nih.gov/pubmed/19245415?ordinalpos=1&itool=EntrezSystem2.PEntrez.Pubmed.Pubmed_ResultsPanel.Pubmed_DefaultReportPanel.Pubmed_RVDocSum)J Am Geriatr Soc. 2009 Mar;57(3):453-61

Boter H, van Delden JJ, de Haan RJ, Rinkel GJ. Modified informed consent procedure: consent to postponed information. BMJ 2003; 327:284-285

Boult, C., Reider, L., Frey, K., Leff, B., Boyd, C. M., Wolff, J. L. et al. (2008). Early effects of "Guided Care" on the quality of health care for multimorbid older persons: a cluster-randomized controlled trial. *J.Gerontol.A Biol.Sci.Med.Sci., 63,* 321-327.

Boyd, C. M., Landefeld, C. S., Counsell, S. R., Palmer, R. M., Fortinsky, R. H., Kresevic, D. et al. (2008). Recovery of activities of daily living in older adults after hospitalization for acute medical illness. *J.Am.Geriatr.Soc., 56,* 2171-2179.

Boyd, C. M., Xue, Q. L., Simpson, C. F., Guralnik, J. M., & Fried, L. P. (2005). Frailty, hospitalization, and progression of disability in a cohort of disabled older women. *Am.J.Med., 118,* 1225-1231.

Campbell, S. E., Seymour, D. G., Primrose, W. R., Lynch, J. E., Dunstan, E., Espallargues, M. et al. (2005). A multi-centre European study of factors affecting the discharge destination of older people admitted to hospital: analysis of in-hospital data from the ACMEplus project. *Age Ageing, 34,* 467-475.

Counsell, S. R., Callahan, C. M., Clark, D. O., Tu, W., Buttar, A. B., Stump, T. E. et al. (2007). Geriatric care management for low-income seniors: a randomized controlled trial. *JAMA, 298,* 2623-2633.

Ellis G, Langhorne P.[Comprehensive geriatric assessment for older hospital patients.](http://www.ncbi.nlm.nih.gov/pubmed/15684245?ordinalpos=4&itool=EntrezSystem2.PEntrez.Pubmed.Pubmed_ResultsPanel.Pubmed_DefaultReportPanel.Pubmed_RVDocSum)

Br Med Bull. 2005 Jan 31;71:45-59. Print 2004. Review.

Eklund K, Wilhelmson K.[Outcomes of coordinated and integrated interventions targeting frail elderly people: a systematic review of randomised controlled trials.](http://www.ncbi.nlm.nih.gov/pubmed/19245421?ordinalpos=1&itool=EntrezSystem2.PEntrez.Pubmed.Pubmed_ResultsPanel.Pubmed_DefaultReportPanel.Pubmed_RVDocSum) Health Soc Care Community. 2009 Feb 24.

Ferrucci, L., Guralnik, J. M., Studenski, S., Fried, L. P., Cutler, G. B., Jr., & Walston, J. D. (2004). Designing randomized, controlled trials aimed at preventing or delaying functional decline and disability in frail, older persons: a consensus report. *J.Am.Geriatr.Soc., 52,* 625-634.

Fried LP, Tangen CM, Walston J, Newman AB, Hirsch C, Gottdiener J, Seeman T, Tracy R, Kop WJ, Burke G, McBurnie MA; Cardiovascular Health Study Collaborative Research Group.[Frailty in older adults: evidence for a phenotype.](http://www.ncbi.nlm.nih.gov/pubmed/11253156?ordinalpos=13&itool=EntrezSystem2.PEntrez.Pubmed.Pubmed_ResultsPanel.Pubmed_DefaultReportPanel.Pubmed_RVDocSum)J Gerontol A Biol Sci Med Sci. 2001 Mar;56(3):M146-56.

Gill, T. M., Hardy, S. E., & Williams, C. S. (2002). Underestimation of disability in community-living older persons. *J.Am.Geriatr.Soc., 50,* 1492-1497.

Graff, M. J., Adang, E. M., Vernooij-Dassen, M. J., Dekker, J., Jonsson, L., Thijssen, M. et al. (2008). Community occupational therapy for older patients with dementia and their care givers: cost effectiveness study. *BMJ, 336,* 134-138.

Gravelle H, Dusheiko M, Sheaff R, Sargent P, Boaden R, Pickard S, Parker S, Roland M.[Impact of case management (Evercare) on frail elderly patients: controlled before and after analysis of quantitative outcome data.](http://www.ncbi.nlm.nih.gov/pubmed/17107984?ordinalpos=8&itool=EntrezSystem2.PEntrez.Pubmed.Pubmed_ResultsPanel.Pubmed_DefaultReportPanel.Pubmed_RVDocSum) BMJ. 2007 Jan 6;334(7583):31.

Hallberg IR, Kristensson J. [Preventive home care of frail older people: a review of recent case management studies.](http://www.ncbi.nlm.nih.gov/pubmed/15724826?ordinalpos=6&itool=EntrezSystem2.PEntrez.Pubmed.Pubmed_ResultsPanel.Pubmed_DefaultReportPanel.Pubmed_RVDocSum)J Clin Nurs. 2004 Sep;13(6B):112-20. Review.

De Haan RJ, Vermeulen M, Holman R, Lindeboom R. Measuring the functional status of

patients in clinical trials using modern clinimetric methods [Dutch]. Ned Tijdschr Geneeskd 2002;146:606-11.

Holman R, Lindeboom R, Glas CAW, Vermeulen M, de Haan RJ. Constructing an item bank using item response theory: the amc linear disability score project. Health services and outcomes research Methodology 2003;4:19-33.

Holman R, Glas CAW, de Haan RJ. Power analysis in randomized clinical trials based on the item response theory. Control clin trials 2003; 24:390-410.

Holman R, Weisscher N, Glas CA, Dijkgraaf MG, Vermeulen M, de Haan RJ, Lindeboom R. The Academic Medical Center Linear Disability Score (ALDS) item bank: item response theory analysis in a mixed patient population. Health Qual Life Outcomes 2005;3:83

Holman R, Glas CAW. Modelling non-ignorable missing data mechanisms with item response theory models. Br J Math Stat Psychol. 2005; 58:1-17.

Huyse FJ, de Jonge P, Slaets JP, Herzog T, Lobo A, Lyons JS, Opmeer BC, Stein B, Arolt V, Balogh N, Cardoso G, Fink P, Rigatelli M. [COMPRI–an instrument to detect patients with complex care needs: results from a European study.](http://www.ncbi.nlm.nih.gov/pubmed/11351110?ordinalpos=7&itool=EntrezSystem2.PEntrez.Pubmed.Pubmed_ResultsPanel.Pubmed_DefaultReportPanel.Pubmed_RVDocSum)Psychosomatics. 2001 May-Jun;42(3):222-8.

Huss A, Stuck AE, Rubenstein LZ, Egger M, Clough-Gorr KM.[Multidimensional preventive home visit programs for community-dwelling older adults: a systematic review and meta-analysis of randomized controlled trials.](http://www.ncbi.nlm.nih.gov/pubmed/18375879?ordinalpos=6&itool=EntrezSystem2.PEntrez.Pubmed.Pubmed_ResultsPanel.Pubmed_DefaultReportPanel.Pubmed_RVDocSum)J Gerontol A Biol Sci Med Sci. 2008 Mar;63(3):298-307. Review.

Landi, F., Onder, G., Cesari, M., Barillaro, C., Lattanzio, F., Carbonin, P. U. et al. (2004). Comorbidity and social factors predicted hospitalization in frail elderly patients. *J.Clin.Epidemiol., 57,* 832-836.

Levine MD, Mahoney LD, Heagerty PJ, Wagner EH. [Bringing geriatricians to the front lines: evaluation of a quality improvement intervention in primary care.](http://www.ncbi.nlm.nih.gov/pubmed/16809646?ordinalpos=8&itool=EntrezSystem2.PEntrez.Pubmed.Pubmed_ResultsPanel.Pubmed_DefaultReportPanel.Pubmed_RVDocSum)J Am Board Fam Med. 2006 Jul-Aug;19(4):331-9.

McCusker, J., Bellavance, F., Cardin, S., Trepanier, S., Verdon, J., & Ardman, O. (1999). Detection of older people at increased risk of adverse health outcomes after an emergency visit: the ISAR screening tool. *J.Am.Geriatr.Soc., 47,* 1229-1237.

Meiland, F. J., Droes, R. M., de, L. J., & Vernooij-Dassen, M. J. (2005). Facilitators and barriers in the implementation of the meeting centres model for people with dementia and their carers. *Health Policy, 71,* 243-253.

Naylor, M. D., Brooten, D., Campbell, R., Jacobsen, B. S., Mezey, M. D., Pauly, M. V. et al. (1999). Comprehensive discharge planning and home follow-up of hospitalized elders: a randomized clinical trial. *JAMA, 281,* 613-620.

Pot, A. M., van, D. R., & Deeg, D. J. (1995). [Perceived stress caused by informal caregiving. Construction of a scale]. *Tijdschr.Gerontol.Geriatr., 26,* 214-219.

Rockwood, K., Hogan, D. B., & MacKnight, C. (2000). Conceptualisation and measurement of frailty in elderly people. *Drugs Aging, 17,* 295-302.

Schellings R. Pre-randomization in study designs. Acceptability and applicability. Thesis, University of Maastricht 2005.

[Sager MA](http://www.ncbi.nlm.nih.gov/sites/entrez?Db=pubmed&Cmd=Search&Term="Sager MA"%5BAuthor%5D&itool=EntrezSystem2.PEntrez.Pubmed.Pubmed_ResultsPanel.Pubmed_DiscoveryPanel.Pubmed_RVAbstractPlus), [Rudberg MA](http://www.ncbi.nlm.nih.gov/sites/entrez?Db=pubmed&Cmd=Search&Term="Rudberg MA"%5BAuthor%5D&itool=EntrezSystem2.PEntrez.Pubmed.Pubmed_ResultsPanel.Pubmed_DiscoveryPanel.Pubmed_RVAbstractPlus), [Jalaluddin M](http://www.ncbi.nlm.nih.gov/sites/entrez?Db=pubmed&Cmd=Search&Term="Jalaluddin M"%5BAuthor%5D&itool=EntrezSystem2.PEntrez.Pubmed.Pubmed_ResultsPanel.Pubmed_DiscoveryPanel.Pubmed_RVAbstractPlus), [Franke T](http://www.ncbi.nlm.nih.gov/sites/entrez?Db=pubmed&Cmd=Search&Term="Franke T"%5BAuthor%5D&itool=EntrezSystem2.PEntrez.Pubmed.Pubmed_ResultsPanel.Pubmed_DiscoveryPanel.Pubmed_RVAbstractPlus), [Inouye SK](http://www.ncbi.nlm.nih.gov/sites/entrez?Db=pubmed&Cmd=Search&Term="Inouye SK"%5BAuthor%5D&itool=EntrezSystem2.PEntrez.Pubmed.Pubmed_ResultsPanel.Pubmed_DiscoveryPanel.Pubmed_RVAbstractPlus), [Landefeld CS](http://www.ncbi.nlm.nih.gov/sites/entrez?Db=pubmed&Cmd=Search&Term="Landefeld CS"%5BAuthor%5D&itool=EntrezSystem2.PEntrez.Pubmed.Pubmed_ResultsPanel.Pubmed_DiscoveryPanel.Pubmed_RVAbstractPlus), [Siebens H](http://www.ncbi.nlm.nih.gov/sites/entrez?Db=pubmed&Cmd=Search&Term="Siebens H"%5BAuthor%5D&itool=EntrezSystem2.PEntrez.Pubmed.Pubmed_ResultsPanel.Pubmed_DiscoveryPanel.Pubmed_RVAbstractPlus), [Winograd CH](http://www.ncbi.nlm.nih.gov/sites/entrez?Db=pubmed&Cmd=Search&Term="Winograd CH"%5BAuthor%5D&itool=EntrezSystem2.PEntrez.Pubmed.Pubmed_ResultsPanel.Pubmed_DiscoveryPanel.Pubmed_RVAbstractPlus). Hospital admission risk profile (HARP): identifying older patients at risk for functional decline following acute medical illness and hospitalization. [Am Geriatr Soc.](javascript:AL_get(this, 'jour', 'J Am Geriatr Soc.');) 1996 Mar;44(3):251-7

Studenski S, Hayes RP, Leibowitz RQ, Bode R, Lavery L, Walston J, Duncan P, Perera S.[Clinical Global Impression of Change in Physical Frailty: development of a measure based on clinical judgment.](http://www.ncbi.nlm.nih.gov/pubmed/15341562?ordinalpos=5&itool=EntrezSystem2.PEntrez.Pubmed.Pubmed_ResultsPanel.Pubmed_DefaultReportPanel.Pubmed_RVDocSum)J Am Geriatr Soc. 2004 Sep;52(9):1560-6

Walston, J., Hadley, E. C., Ferrucci, L., Guralnik, J. M., Newman, A. B., Studenski, S. A. et al. (2006). Research agenda for frailty in older adults: toward a better understanding of physiology and etiology: summary from the American Geriatrics Society/National Institute on Aging Research Conference on Frailty in Older Adults. *J.Am.Geriatr.Soc., 54,* 991-1001.

Walters, K., Iliffe, S., Tai, S. S., & Orrell, M. (2000). Assessing needs from patient, carer and professional perspectives: the Camberwell Assessment of need for Elderly people in primary care. *Age Ageing, 29,* 505-510.

Weisscher N, Wijbrandts CA, De Haan R, Glas CAW, Vermeulen M, Tak PP. The academic medical center linear disability score item bank: psychometric properties of a new generic disability measure in rheumatoid arthritis. J Rheumatol. 2007;34:1222-8

Weisscher N, Post B, De Haan RJ, Glas CAW, Speelman JD, Vermeulen M. The AMC linear disability score in patients with newly diagnosed Parkinson disease. Neurology 2007;69:2155-61.

APPENDIX 1: Diagnostic Comprehensive Geriatric Assessment (versie juni 2010)

|  |  |  |
| --- | --- | --- |
| **Domeinen** | **Samenvattingsvel CGA** | **Problemen/**  **Ziekten** |
| **SOMATISCH** | | |
| 1. Mobiliteit en  Stabiliteit | Q1 Bent u het afgelopen jaar 1 of meerdere keren gevallen?  Q2 Heeft u last van duizeligheid? Zo ja, heeft het invloed op uw dagelijk leven?  Q3 Hebt u ooit iets gebroken? Zo ja, FRAX score | Vallen/ fractuurrisico  Duizeligheid  Osteoporose |
| 2. Medicatie | Alleen bij medicatiegebruik  Q4 Ervaart u problemen en/of bijwerkingen  Q5 Registratie gebruik van 5 of meer verschillende soorten medicatie  Q6 Vragenlijst van Aburuz | Veiligheid en bijwerkingen |
|  |  | Polyfarmacie |
|  |  | Therapietrouw |
| 3.Voeding | Q7 SNAQ-vragenlijst  Q8 Is sprake van uitdrogingsverschijnselen?  Q9 Heeft u moeite met slikken?  Q11 BMI  Q10 Heeft u pijn in uw mond? | Ondervoeding |
|  |  | Dehydratie |
|  |  | Slikstoornis |
|  |  | Obesitas |
|  |  | Mondhygiëne |
| 4. Mictie en defecatie problemen | Q12 Bent u incontinent van urine? Bent u incontinent voor ontlasting  Q13Heeft u last van obstipatie?  Q14 Heeft u een blaascatheter ? | Incontinentie |
|  |  | Obstipatie |
|  |  | Blaaskatheter |
| 5. Huid | Q15. Heeft u decubitus/doorligplekken? | Decubitus/ Wonden |
| 6. Pijn | Q16. VAS score of Painad | Pijn |
| 7. Allergie | Q17.Heeft u last van allergie/ allergische reactie? | Allergie |

| PSYCHISCH |  |  |
| --- | --- | --- |
| 1.Delirium | Q18 Eerder doorgemaakt delier ?  Q19. Confusement Assessment Method | Delirium |
| 2. Depressie | Q20. Geriatric depression Scale, bij positieve GDS-3: GDS-15 | Depressie  En Rouw |
| 3.Cognitie | Q21. Minimal Mental State Examination | Cognitieve stoornis |
| 4. Angst | Q22 Voelt u zich wel eens angstig van binnen? | Angststoornis |
| 5. Afhankelijkheid | Q23a roken  Q24b alcohol  Q25c Gebruikt u kalmeringsmiddelen ?  Zoja welke en aantal per week. | genotsmiddelen en medicatie |

| FUNCTIONEEL |  |  |
| --- | --- | --- |
| 1. ADL en IADL functioneren | Q26 a Gemodificeerde Katz, ADL vragen | ADL afhankelijkheid |
|  | Q27 b Gemodificeerde Katz, IADL vragen | IADL afhankelijkheid |
|  | Q28c Gebruikt u een loophulpmiddel? | Mobiliteit en transfer |
| 2. Slechthorendheid | Q29 Heeft u problemen met horen, ondanks gebruik apparaten? | Slechthorendheid |
| 3. Slechtziendheid | Q30 Heeft u problemen met zien, ondanks gebruik van bril? | Slechtziendheid |
| 4. Slaap | Q31a Ervaart u problemen met slapen?  Q32b Gebruikt u wel eens slaapmedicatie?zo ja, hoe vaak? | Slaapstoornis |

| SOCIAAL |  |  |
| --- | --- | --- |
| 1. Sociale participatie | Q33. De Jong Gierveld-schaal | Eenzaamheid en sociale participatie |
| 2. Woonsituatie | Q34.Voldoet uw huidige woonomgeving aan uw wensen? | Problematische woonsituatie |
| 3. Beperkte financiën | Q35. Ervaart u momenteel financiële problemen? | Financiële problemen |
| 4. Kwaliteit van leven | Q36.Hoe is in het algemeen uw kwaliteit van leven?  Q37. Welk rapportcijfer geeft u uw leven op dit moment?  Q38.Hoe is in het algemeen uw kwaliteit van leven, in vergelijking met een jaar geleden?  Q39. EQ-6D | Kwaliteit van Leven |
| 5.Gezondheid en welbevinden | Q40. Hoe is in het algemeen uw gezondheid? (Q35)  Q41. Hoe is in het algemeen uw gezondheid, in vergelijking met een jaar geleden? | Ervaren gezondheid |

APPENDIX 2: POSTPONED INFORMED CONSENT PROCEDURE

Let op:

De studie maakt gebruik van een ‘***informed consent voor uitgestelde informatie-procedure’***; patiënten weten ***niet*** dat er twee verschillende behandelingen zijn en dat ze gerandomiseerd worden.

HIS General Practice Information System

Inclusiecriteria:

- Leeftijd ≥70 jaar
- Thuiswonend
- Beheersing van de Nederlandse taal
- Niet terminaal ziek of dementerend

**⇩**

Schriftelijke enquete voor alle 70+ ers van zowel interventie als controle groepen.

In schriftelijke enquete, zit:

- Informed consent
- informatiebrief
- ISAR-PC lijst en Minimale Data Set

Patiënt vult formulieren in met ISAR-PC vragen en Informed Consent en stuurt die retour naar het AMC.

**⇩**

*Uitleg:*

In deze studie onderzoeken we 2 zaken:

1. Of er het mogelijk is om (verder) functieverlies bij ouderen te beperken of te voorkomen.
2. Over de tweede onderzoeksvraag kunnen wij u op dit moment niet informeren, omdat dit de onderzoeksresultaten kan beïnvloeden. We zullen u na afloop van het onderzoek over dit aspect informeren.

Algemeen: De medisch ethische toetsingscommissie van het ziekenhuis heeft deze studie goedgekeurd. Er zijn geen risico’s aan de studie verbonden.

**⇩**

POH neemt CGA af bij patiënten met verhoogd risico in interventiegroep.

*Interventiegroep:* praktijkondersteuner ouderenzorg

*Controlegroep:* gebruikelijke zorg: ´care as usual’

**⇩**

Interventiegroep: POH bespreekt met de patiënten de uitkomsten van het diagnostisch assessment en bespreekt welke geriatrische problemen volgens patiënt aangepakt moeten worden.

**⇩**

Interventiegroep: POH overlegt met de huisarts over de uitkomsten van het CGA en het zorgbehandelplan.

**⇩**

Interventiegroep: POH start en coördineert indien nodig aanvullende behandelingen.

**⇩**

Interventiegroep: POH vervolgt de patiënt gedurende de studieperiode.
