# Supplementary material for: Effects of Nurse-Led Multifactorial Care to Prevent Disability in Community-Living Older People: Cluster Randomized Trial
Source: PLoS One. 2016 Jul 26;11(7):e0158714. doi: 10.1371/journal.pone.0158714 (PMC4961429; doi:10.1371/journal.pone.0158714)
Supplement: S1 Table — (DOC) [file pone.0158714.s006.doc]

## S1 Table: Characteristics of participants and general practices in the intervention group

| **General practice** | **Intervention group** | | | | | | | | | | |
| --- | --- | --- | --- | --- | --- | --- | --- | --- | --- | --- | --- |
|  | **1** | **2** | **3** | **4** | **5** | **6** | **7** | **8** | **9** | **10** | **11** |
| Participants (n) | 131 | 143 | 74 | 89 | 99 | 46 | 228 | 177 | 31 | 113 | 78 |
| Age, in years,  median (IQR) | 83.5  (78.3-87.3) | 82.5  (76.5-87.2) | 83.1  (77.1-87.1) | 85.4  (79.8-90.0) | 82.6  (76.4-87.1) | 78.5  (73.9-84.9) | 82.1  (76.2-86.8) | 83.3  (78.9-86.9) | 84.0  (76.7-88.1) | 82.6  (76.9-88.0) | 84.1  (75.9-87.9) |
| female sex | 68.7 | 59.4 | 62.2 | 69.7 | 65.7 | 63.0 | 64.5 | 68.4 | 61.3 | 65.5 | 62.8 |
| Socio-economic status   low (≤1SD)  intermediate  high ≥1SD) | 1.5 97.7 0.8 | - 100 - | 1.4 98.6 - | 1.1 98.9 - | 1.0 97.0 2.0 | - 95.7 4.3 | - 51.8 48.2 | - 55.9 44.1 | - 100 - | 22.1 51.3 26.5 | 35.9 62.8 1.3 |
| Katz-ADL (range 0-6),  median (IQR) | 1 (0-1) | 1 (0-1) | 0 (0 -1) | 1 (0-2) | 1 (0-2) | 1 (0-1) | 0 (0-1) | 1 (0-1) | 1 (1-2) | 1 (0-1) | 1 (0 -1) |
| IADL scale (range 0-7), median (IQR) | 1 (1-3) | 1 (0-3) | 1 (0-2) | 2 (1-4) | 2 (0-3) | 1 (1-3) | 1 (0-2) | 2 (1-3) | 2.0 (1-4) | 2 (0-3) | 1 (0-3) |

Values are numbers (percentages) unless stated otherwise; IQR=interquartile range; SD=standard deviation; Katz-activities of daily living; IADL=instrumental activities of daily living.
