# Supplementary material for: Effects of Nurse-Led Multifactorial Care to Prevent Disability in Community-Living Older People: Cluster Randomized Trial
Source: PLoS One. 2016 Jul 26;11(7):e0158714. doi: 10.1371/journal.pone.0158714 (PMC4961429; doi:10.1371/journal.pone.0158714)
Supplement: S2 Table — (DOC) [file pone.0158714.s007.doc]

## S2 Table: Characteristics of participants and general practices in the control group

| **General practice** | **control group** | | | | | | | | | | | | |
| --- | --- | --- | --- | --- | --- | --- | --- | --- | --- | --- | --- | --- | --- |
|  | **1** | **2** | **3** | **4** | **5** | **6** | **7** | **8** | **9** | **10** | **11** | **12** | **13** |
| Participants (n) | 102 | 38 | 60 | 70 | 84 | 188 | 151 | 69 | 19 | 85 | 59 | 83 | 66 |
| Age, in years,  median (IQR) | 84.6  (79.5-88.0) | 81.6  (76.8-86.5) | 80.4  (74.7-85.7) | 82.6  (75.6-86.5) | 83.9  (77.2-87.4) | 82.6  (77.7-86.5) | 83.0  (76.8-87.8) | 81.0  (75.0-86.6) | 77.5  (74.1-82.9) | 81.3  (75.9-86.6) | 81.9  (76.6-85.3) | 83.2  (77.2-86.8) | 80.7  (74.9-86.8) |
| female sex | 56.9 | 73.7 | 61.7 | 62.9 | 63.1 | 64.4 | 57.6 | 60.9 | 73.7 | 62.4 | 66.1 | 63.9 | 66.7 |
| Socio-economic status   low (≤1SD)  intermediate  high ≥1SD) | - 99.0 1.0 | 2.6 97.4 - | - 100 - | - 77.1 22.9 | 1.2 36.9 61.9 | - 98.9 1.1 | 10.6 86.1 3.3 | - 91.3 8.7 | - 63.2 36.8 | - 98.8 1.2 | 47.5 49.2 3.4 | 27.7 69.9 2.4 | 13.6 74.2 12.1 |
| Katz-ADL (range 0-6), median (IQR) | 0 (0-1) | 1 (0-2) | 1 (0-1) | 1 (0-1) | 0 (0-1) | 1 (0-2) | 1 (0-2) | 0 (0-1) | 1 (0-1) | 0 (0-1) | 1 (0-1) | 1 (0-1) | 1 (0-1) |
| IADL scale (range 0-7), median (IQR) | 1 (0-3) | 2 (0-3) | 1 (0-3) | 1 (1-3) | 1 (0-3) | 2 (1-4) | 2 (1-4) | 1 (0-3) | 2 (1-3) | 1 (0-0) | 2 (1-3) | 2 (0-4) | 1 (0-3) |

Values are numbers (percentages) unless stated otherwise; IQR=interquartile range; SD=standard deviation; Katz-activities of daily living; IADL=instrumental activities of daily living.
