# Supplementary material for: Effects of Nurse-Led Multifactorial Care to Prevent Disability in Community-Living Older People: Cluster Randomized Trial
Source: PLoS One. 2016 Jul 26;11(7):e0158714. doi: 10.1371/journal.pone.0158714 (PMC4961429; doi:10.1371/journal.pone.0158714)
Supplement: S3 Table — (DOC) [file pone.0158714.s008.doc]

## S3 Table: Characteristics of participants in the intervention group who received or declined the comprehensive geriatric assessment

| **Characteristics** | **Intervention group**  **received CGA**  **N= 934** | **Intervention group  declined CGA**  **N= 275** |
| --- | --- | --- |
|  | **n(%)** | **%** |
| Age, in years, median (IQR) | 82.7 (76.8-87.1) | 84.0* (78.2.6-88.1) |
| Female sex | 65.4 | 64.2 |
| Level of education  primary school or less  secondary education  college or university | 20.3  64.0  15.7 | 24.7  62.5  12.7 |
| Socio-economic status  low (≥1SD)  intermediate  high (≤1SD) | 4.3  77.4  18.3 | 6.5  74.2  19.4 |
| Married/living together | 47.1 | 45.5 |
| Living situation  independent, alone  independent, together  home for elderly | 46.2  44.6  8.2 | 36.7***  41.0  22.3 |
| Multimorbidity (≥2) | 83.4 | 82.3 |
| Polypharmacy (≥3)  Memory problems (self report) | 69.0  33.3 | 71.0  38.7 |
| Modified Katz-ADL index (range 0-15), median (IQR)  Katz-ADL (range 0-6), median (IQR)  IADL scale (range 0-7), median (IQR) | 2.0 (1-4)  1.0 (0-1)  1.0 (0-3) | 3.0*** (1-6)  1.0*** (0-1)  2.0*** (1-4) |
| EuroQol-5D (range -0.33 to 1.0), mean (SD) | 0.76 (0.20) | 0.70*** (0.25) |
| Emotional wellbeing (RAND36), (range 4-100), mean (SD) | 72.2 (16.9) | 68.5** (18.5) |
| Quality of Life mark (range 0-10), mean (SD) | 7.3 (1.2) | 6.9*** (1.5) |
| Falls (≥1) in past 12 months | 34.6 | 36.0 |

Values are numbers (percentages) unless stated otherwise; IQR=interquartile range; SD=standard deviation; Katz-activities of daily living; IADL=instrumental activities of daily living.

Student’s t-test for continuous variables; Mann-Whitney U test for continuous nonparametric variables; Chi-square test for categorical variables.

*p<0.05; **p<0.01; ***p<0.001
