# Supplementary material for: Effects of Nurse-Led Multifactorial Care to Prevent Disability in Community-Living Older People: Cluster Randomized Trial
Source: PLoS One. 2016 Jul 26;11(7):e0158714. doi: 10.1371/journal.pone.0158714 (PMC4961429; doi:10.1371/journal.pone.0158714)
Supplement: S4 Table — (DOC) [file pone.0158714.s009.doc]

## S4 Table: Prevalence of geriatric conditions in CGA

| **Geriatric condition** | **Description** | **%** |
| --- | --- | --- |
| Mobility problems | Use of walking aid | 47.2 |
| Polypharmacy (≥5) | Use ≥5 different medications | 45.5 |
| Blood pressure | Systolic blood pressure ≥ 160 mmHg | 43.4 |
| Sleeping disorder | Problems with sleeping or use of sleeping medication | 39.6 |
| Physical inactivity | Physical activity < once a month | 26.1 |
| Falls | At least one fall (in past 12 months) | 38.4 |
| Depression | Geriatric depression scale (one of two questions) | 38.1 |
| Pain | Score ≥ 4 on visual analogue scale | 37.7 |
| Urine incontinence | Involuntary loss of urine | 37.1 |
| Cognitive impairment | Self-report memory problem | 35.3 |
| Loneliness | Score ≥ 3 on Jong Gierveld loneliness scale | 32.7 |
| Osteoporosis risk | Score ≥ 4 on osteoporosis risk factors | 28.1 |
| Hearing impairment | Self-report hearing impairment | 27.3 |
| Alcohol use | Score: ≥ 4 units/week women; ≥ 5 units/week men | 24.2 |
| Medication use | Problems with compliance or side-effects | 22.0 |
| Obesity | BMI > 30 kg/m2 | 18.6 |
| Visual impairment | Self-report visual impairment | 18.3 |
| Dizziness | Problems of dizziness in the last month | 13.2 |
| Anxiety | Feelings of anxiety (in the last month) | 10.4 |
| Oral hygiene | Problems or pain of mouth in last month | 9.2 |
| Living situation | inadequate living situation | 7.8 |
| Constipation | Stool < 3 times a week | 5.5 |
| MMSE | Score ≤ 23 mini mental state examination | 4.4 |
| Malnutrition | SNAQ 65+ | 3.1 |

BMI = Body Mass Index; SNAQ = Short Nutritional Assessment Questionnaire.
