# Supplementary material for: Effects of Nurse-Led Multifactorial Care to Prevent Disability in Community-Living Older People: Cluster Randomized Trial
Source: PLoS One. 2016 Jul 26;11(7):e0158714. doi: 10.1371/journal.pone.0158714 (PMC4961429; doi:10.1371/journal.pone.0158714)
Supplement: S5 Table — (DOC) [file pone.0158714.s010.doc]

## S5 Table: Adherence to the trial protocol

| **Participants in intervention n = 1209** | **% (n/N)** |
| --- | --- |
| Comprehensive geriatric assessment | 77.0 (934/1209) |
| Care and treatment plan (CTP) | 76.6 (926 /1209) |
| CTP discussed with General Practitioner | 61.6 (575/934) |
| Evaluation of CTP after one year | 77.4 (698/898*) |
| Decline CTP: participants declining care | 16.9(158/936) |
| Decline CTP: problems already being addressed | 14.2 (133/936) |
| Decline CTP: participants not recognizing the identified problem | 7.3 (68/936) |

Values are numbers (percentages) unless stated otherwise.
*after one year 38 persons died.
