# Supplementary material for: Effects of Nurse-Led Multifactorial Care to Prevent Disability in Community-Living Older People: Cluster Randomized Trial
Source: PLoS One. 2016 Jul 26;11(7):e0158714. doi: 10.1371/journal.pone.0158714 (PMC4961429; doi:10.1371/journal.pone.0158714)
Supplement: S6 Table — (DOC) [file pone.0158714.s011.doc]

## S6 Table: Primary results of trial: Mean scores and difference between intervention and control arm

## at 6, 12, 18 and 24 months

| **Outcome** | **6 months** | | **12 months** | | **18 months** | | | **24 months** | | **6, 12, 18, 24 months** | |
| --- | --- | --- | --- | --- | --- | --- | --- | --- | --- | --- | --- |
|  | **Mean score**  **(95% CI)** | | **Mean score**  **(95% CI)** | | **Mean score**  **(95% CI)** | | | **Mean score**  **(95% CI)** | | **Mean difference (95% CI)** | **p-value** |
|  | **Intervention** | **Control** | **Intervention** | **Control** | **Intervention** | **Control** | **Intervention** | | **Control** | **Time X treatment (p_interaction_=0.68).** | |
| Modified Katz-ADL index (0-15) * | 3.11  (2.94-3.28) | 3.32  (3.13-3.50) | 3.38  (3.20-3.56) | 3.59  (3.39-3.78) | 3.53  (3.34-3.71) | 3.74  (3.54-3.93) | 3.27  (3.09-3.45) | | 3.48  (3.28-3.67) | -0.21  (-0.46-0.04) | 0.10 |
|  |  |  |  |  |  |  |  | |  |  |  |
| Modified Katz-ADL index (0-15) ** | 3.05  (2.94-3.15) | 3.10  (2.98-3.21) | 3.33  (3.21-3.44) | 3.38  (3.25-3.51) | 3.47  (3.35-3.59) | 3.52  (3.39-3.65) | 3.20  (3.07-3.34) | | 3.26  (3.11-3.40) | -0.05  (-0.20-0.10) | 0.49 |
|  |  |  |  |  |  |  |  | |  |  |  |
| Modified Katz-ADL index (0-15)  *** | 3.02  (2.92-3.12) | 3.09  (2.98-3.21) | 3.31  (3.20-3.42) | 3.39  (3.26-3.51) | 3.46  (3.33-3.58) | 3.53  (3.40-3.66) | 3.19  (3.05-3.32) | | 3.27  (3.12-3.41) | -0.07  (-0.22-0.07) | 0.33 |

* Estimated mean scores and mean differences between intervention and control arm (unadjusted).

** Estimated mean scores and mean difference between intervention and control arm adjusted for baseline modified Katz-ADL index score, which was selected on the basis of causal diagrams.
*** Estimated mean scores and mean difference between intervention and control arm adjusted for baseline variables, which were selected on the basis of causal diagrams. Analysis was adjusted for age, sex, socio-economic status, level of education, and modified Katz-ADL index score.
CI = confidence interval. The mean difference was the same at all follow-up moments.
