# Supplementary material for: Effects of Nurse-Led Multifactorial Care to Prevent Disability in Community-Living Older People: Cluster Randomized Trial
Source: PLoS One. 2016 Jul 26;11(7):e0158714. doi: 10.1371/journal.pone.0158714 (PMC4961429; doi:10.1371/journal.pone.0158714)
Supplement: S7 Table — (DOC) [file pone.0158714.s012.doc]

S7 Table: Primary results of trial: Mean difference between intervention and control group at 12 months after accounting for missing values

| **Outcome** | **No of participants in MLA** | **Mean difference**  **(95% CI)** | **p-value** |
| --- | --- | --- | --- |
| Modified Katz-ADL index *** | 2249 | -0.05  (-0.20-0.10) | 0.52 |

*** Estimated mean scores and mean difference between intervention and control arm adjusted for baseline variables, which were selected on the basis of causal diagrams. Analysis was adjusted for age, sex, socio-economic status, level of education, and modified Katz-ADL index score.
CI = confidence interval, MLA = multilevel analyses.
Data were based on 10 imputed datasets.
