# Supplementary material for: Effects of Nurse-Led Multifactorial Care to Prevent Disability in Community-Living Older People: Cluster Randomized Trial
Source: PLoS One. 2016 Jul 26;11(7):e0158714. doi: 10.1371/journal.pone.0158714 (PMC4961429; doi:10.1371/journal.pone.0158714)
Supplement: S8 Table — (DOC) [file pone.0158714.s013.doc]

## S8 Table: Mean scores and differences between intervention and control group at 6, 12, 18 and 24 months for secondary outcomes health related quality of life and emotional wellbeing

| **Outcome** | **6 months** | | | **12 months** | | | **18 months** | | | | **24 months** | | |
| --- | --- | --- | --- | --- | --- | --- | --- | --- | --- | --- | --- | --- | --- |
|  | **Mean score (95% CI)** | | **Mean difference (95% CI),**  **p-value** | **Mean score (95% CI)** | | **Mean difference (95% CI),**  **p-value** | **Mean score (95% CI)** | | | **Mean difference**  **(95% CI),**  **p-value** | **Mean score (95% CI)** | | **Mean difference (95% CI),**  **p-value** |
|  | **Intervention** | **Control** |  | **Intervention** | **Control** |  | | **Intervention** | **Control** |  | **Intervention** | **Control** |  |
| EQ5D* | 0.76  (0.75-0.77) | 0.75  (0.73-0.76) | 0.01  (-0.01-0.03),  0.20 | 0.74  (0.72-0.75) | 0.72  (0.71-0.74) | 0.01  (-0.01-0.03),  0.17 | | 0.75  (0.72-0.76) | 0.71  (0.69-0.73) | 0.03  (0.01-0.05),  0.01 | 0.73  (0.71-0.74) | 0.71  (0.69-0.72) | 0.02  (0.00-0.04),  0.03 |
|  |  |  |  |  |  |  | |  |  |  |  |  |  |
| EQ5D** | 0.76  (0.75-0.77) | 0.76  (0.75-0.77) | -0.003  (-0.02-0.01),  0.70 | 0.74  (0.73-0.75) | 0.74  (0.72-0.75) | 0.00  (-0.02-0.02),  0.97 | | 0.74  (0.73-0.75) | 0.72  (0.71-0.74) | 0.01  (0.00-0.03),  0.11 | 0.73  (0.72-0.74) | 0.72  (0.71-0.73) | 0.01  (-0.01-0.03),  0.33 |
|  |  |  |  |  |  |  | |  |  |  |  |  |  |
| EQ5D*** | 0.76  (0.75-0.77) | 0.76  (0.75-0.77) | 0.00  (-0.02-0.01),  0.72 | 0.74  (0.73-0.75) | 0.74  (0.72-0.75) | 0.00  (-0.01-0.02),  0.84 | | 0.74  (0.73-0.75) | 0.72  (0.71-0.74) | 0.01  (0.00-0.03),  0.12 | 0.73  (0.72-0.74) | 0.72  (0.71-0.73) | 0.01  (-0.01-0.03),  0.29 |
|  |  |  |  |  |  |  | |  |  |  |  |  |  |
| RAND-36 * | 70.25  (70.20-72.29) | 70.39  (70.31-72.48) | -0.15  (-1.65-1.36),  0.85 | 70.08  (68.98-71.19) | 70.33  (69.16-71.50) | -0.25  (-1.86-1.36),  0.77 | | 70.24  (69.12-71.35) | 69.94  (68.49-70.80) | 0.59  (-1.01-2.20),  0.47 | 69.80  (68.67-70.92) | 68.90  (67.71-70.09) | 0.89  (-0.74-2.53),  0.28 |
|  |  |  |  |  |  |  | |  |  |  |  |  |  |
| RAND-36 ** | 71.25  (70.21-72.29) | 71.39  (70.30-72.47) | -0.14  (-1.64-1.37),  0.86 | 70.09  (68.98-71.19) | 70.32  (69.15-71.49) | -0.24  (-1.84-1.37),  0.77 | | 70.24  (69.13-71.35) | 69.63  (68.48-70.79) | 0.61  (-0.99-2.21),  0.46 | 69.80  (68.68-70.93) | 68.89  (67.70-70.08) | 0.90  (-0.73-2.55),  0.28 |
|  |  |  |  |  |  |  | |  |  |  |  |  |  |
| Rand-36 *** | 70.12  (70.07-72.16) | 71.50  (70.42-72.59) | -0.39  (-1.91-1.13),  0.62 | 69.90  (68.79-71.01) | 70.43  (69.26-71.60) | -0.53  (-2.16-1.09),  0.52 | | 70.14  (69.01-71.26) | 69.80  (68.64-70.96) | 0.34  (-1.29-1.97),  0.69 | 69.76  (68.63-70.90) | 69.06  (67.87-70.25) | 0.70  (-0.95-2.36),  0.40 |
|  |  |  |  |  |  |  | |  |  |  |  |  |  |
| QOL* | 7.12  (7.05-7.19 ) | 7.17  (7.09-7.24) | -0.05  (-0.15-0.06),  0.39 | 7.01  (6.93-7.10) | 7.02  (6.94-7.11) | -0.01  (-0.13-0.11),  0.87 | | 6.98  (6.91-7.06) | 6.97  (6.89-7.05) | 0.01  (-0.10-0.13),  0.81 | 6.98  (6.91-7.06) | 6.92  (6.83-7.01) | 0.07  (-0.05-0.18),  0.27 |
|  |  |  |  |  |  |  | |  |  |  |  |  |  |
| QOL** | 7.14  (7.08-7.20) | 7.21  (7.16-7.27) | -0.07  (-0.15-0.01),  0.08 | 7.04  (6.97-7.11) | 7.07  (6.99-7.15) | -0.03  (-0.13-0.08),  0.62 | | 7.01  (6.94-7.07) | 7.02  (6.95-7.10) | -0.02  (-0.11-0.08),  0.73 | 7.01  (6.95-7.07) | 6.98  (6.90-7.06) | 0.03  (-0.07-0.13),  0.52 |
|  |  |  |  |  |  |  | |  |  |  |  |  |  |
| QOL*** | 7.15  (7.09-7.21) | 7.21  (7.15-7.27) | -0.06  (-0.14-0.02),  0.14 | 7.05  (6.98-7.11) | 7.07  (7.00-7.15) | -0.03  (-0.13-0.08),  0.61 | | 7.01  (6.95 -7.08) | 7.03  (6.96 -7.10) | -0.02  (-0.11-0.09),  0.75 | 7.02  (6.96 -7.08) | 6.98  (6.90-7.06) | 0.04  (-0.06-0.15),  0.40 |

* Estimated mean scores and mean differences between intervention and control arm.
** Estimated mean scores and mean differences between intervention and control arm adjusted for baseline outcome.

*** Estimated mean scores and mean difference between intervention and control arm adjusted for baseline variables, which were selected on the basis of causal diagrams. Analysis was adjusted for age, sex, socio-economic status, level of education, and modified Katz-ADL index score.
EQ5D ranges from -0.33 to 1.0 and higher scores indicate better health related quality of life. Rand-36 = emotional wellbeing ranges from 4 to 100 and higher scores indicate better emotional wellbeing. Quality of life mark ranges from 1 to 10 and higher scores indicate better quality of life. CI = confidence interval, QOL = Quality of life.
