# Supplementary material for: Effects of Nurse-Led Multifactorial Care to Prevent Disability in Community-Living Older People: Cluster Randomized Trial
Source: PLoS One. 2016 Jul 26;11(7):e0158714. doi: 10.1371/journal.pone.0158714 (PMC4961429; doi:10.1371/journal.pone.0158714)
Supplement: S9 Table — (DOC) [file pone.0158714.s014.doc]

## S9 Table: Incidence rates and rate ratio’s for intervention and control group at 6, 12, 18 and 24 months for secondary outcomes hospitalization and falls

| **Outcome** | **6 months** | | **12 months** | | **18 months** | | **24 months** | |  | **6, 12, 18, 24 months** | |
| --- | --- | --- | --- | --- | --- | --- | --- | --- | --- | --- | --- |
|  | **Incidence rate (95% CI)** | | **Incidence rate (95% CI)** | | **Incidence rate (95% CI)** | | **Incidence rate (95% CI)** | | **No of participants in MLA** | **Incidence rate ratio (95% CI)** | **p-value** |
|  | **Intervention** | **Control** | **Intervention** | **Control** | **Intervention** | **Control** | **Intervention** | **Control** |  |  |  |
| Hospitalisation  * | 0.10  (0.08-0.11) | 0.11  (0.09-0.13) | 0.10  (0.08-0.12) | 0.11  (0.09-0.13) | 0.09  (0.08-0.11) | 0.10  (0.08-0.12) | 0.10  (0.08-0.12) | 0.11  (0.09-0.14) | 2013 | 0.92  (0.77-1.09) | 0.33 |
|  |  |  |  |  |  |  |  |  |  |  |  |
| Hospitalisation  ** | 0.10  (0.08-0.12) | 0.11  (0.09-0.14) | 0.11  (0.09-0.13) | 0.12  (0.10-0.14) | 0.10  (0.08-0.12) | 0.12  (0.09-0.14) | 0.11  (0.09-0.13) | 0.13  (0.10-0.15) | 1949 | 0.88  (0.74-1.05) | 0.17 |
|  |  |  |  |  |  |  |  |  |  |  |  |
| Hospitalisation  *** | 0.11  (0.09-0.13) | 0.12  (0.10-0.14) | 0.11  (0.09-0.13) | 0.12  (0.10-0.14) | 0.11  (0.09-0.13) | 0.12  (0.10-0.14) | 0.12  (0.09-0.14) | 0.13  (0.10-0.15) | 1924 | 0.90  (0.76-1.07) | 0.25 |
|  |  |  |  |  |  |  |  |  |  |  |  |
| Falls* | 0.20  (0.17-0.23) | 0.17  (0.14-0.20) | 0.20  (0.17-0.23) | 0.17  (0.14-0.19) | 0.21  (0.18-0.25) | 0.18  (0.15-0.21) | 0.21  (0.18-0.25) | 0.18  (0.15-0.21) | 2003 | 1.17  (0.99-1.38) | 0.06 |
|  |  |  |  |  |  |  |  |  |  |  |  |
| Falls ** | 0.25  (0.21-0.29) | 0.22  (0.18-0.26) | 0.25  (0.21-0.29) | 0.22  (0.19-0.26) | 0.29  (0.24-0.33) | 0.25  (0.21-0.29) | 0.28  (0.24-0.33) | 0.25  (0.21-0.29) | 1941 | 1.14  (0.98-1.32) | 0.10 |
|  |  |  |  |  |  |  |  |  |  |  |  |
| Falls*** | 0.25  (0.21-0.29) | 0.22  (0.18-0.26) | 0.26  (0.21-0.30) | 0.22  (0.19-0.26) | 0.29  (0.24-0.34) | 0.25  (0.21-0.30) | 0.29  (0.24-0.34) | 0.25  (0.21-0.30) | 1916 | 1.15  (0.98-1.34) | 0.08 |

* Estimated incidence rates and incidence rate ratio between intervention and control arm
** Estimated incidence rates and incidence rate ratio between intervention and control arm adjusted for baseline score of outcome.
*** Estimated incidence rates and incidence rate ratio between intervention and control arm adjusted for baseline age, sex, socio-economic status, level of education, and score of outcome.

Incidence rate ratios below 1 indicate a protective effect of the intervention. CI = confidence interval. The incidence rate ratios at 12 months were the same at all follow-up moments.
