# Supplementary material for: Effects of Nurse-Led Multifactorial Care to Prevent Disability in Community-Living Older People: Cluster Randomized Trial
Source: PLoS One. 2016 Jul 26;11(7):e0158714. doi: 10.1371/journal.pone.0158714 (PMC4961429; doi:10.1371/journal.pone.0158714)
Supplement: S10 Table — (DOC) [file pone.0158714.s015.doc]

## S10 Table: Incidence rates and rate ratio’s for intervention and control group at 6, 12, 18 and 24 months for secondary outcome after-hours primary care

| **Outcome** | **6 months** | | | | **12 months** | | | **18 months** | | | **24 months** | | |
| --- | --- | --- | --- | --- | --- | --- | --- | --- | --- | --- | --- | --- | --- |
|  | **Incidence rate (95% CI)** | | | **Incidence**  **rate ratio**  **(95% CI),**  **p-value** | **Incidence rate (95% CI)** | | **Incidence**  **rate ratio**  **(95% CI),**  **p-value** | **Incidence rate (95% CI)** | | **Incidence**  **rate ratio**  **(95% CI),**  **p-value** | **Incidence rate (95% CI)** | | **Incidence**  **rate ratio**  **(95% CI),**  **p-value** |
|  | **Intervention** | **Control** |  | | **Intervention** | **Control** |  | **Intervention** | **Control** |  | **Intervention** | **Control** |  |
| After-hours  GP care* | 0.04  (0.03-0.05) | 0.07  (0.05-0.09) | 0.54  (0.38-0.79),  <0.001 | | 0.05  (0.03-0.06) | 0.06  (0.04-0.08) | 0.84  (0.57-1.22),  0.35 | 0.04  (0.03-0.06) | 0.06  (0.04-0.08) | 0.73  (0.49-1.08),  0.12 | 0.06  (0.04-0.08) | 0.06  (0.04-0.08) | 0.98  (0.67-1.45),  0.94 |
|  |  |  |  | |  |  |  |  |  |  |  |  |  |
| After-hours  GP care ** | 0.04  (0.03-0.05) | 0.08  (0.05-0.10) | 0.51  (0.35-0.74),  <0.00 | | 0.06  (0.04-0.08) | 0.06  (0.04-0.09) | 0.87  (-0.60-1.28),  0.49 | 0.05  (0.03-0.06) | 0.07  (0.04-0.09) | 0.72  (-0.48-1.08),  0.11 | 0.07  (0.05-0.10) | 0.07  (0.05-0.10) | 0.97  (0.66-1.43),  0.89 |
|  |  |  |  | |  |  |  |  |  |  |  |  |  |
| After-hours  GP care *** | 0.04  (0.03-0.06) | 0.08  (0.06-0.11) | 0.53  (0.36-0.77),  <0.00 | | 0.06  (0.04-0.08) | 0.06  (0.04-0.08) | 0.95  (0.65-1.41),  0.83 | 0.05  (0.03-0.07) | 0.07  (0.05-0.09) | 0.76  (0.50-1.14),  0.18 | 0.08  (0.05-0.10) | 0.08  (0.05-0.10) | 1.02  (0.69-1.51),  0.90 |

* Estimated incidence rates and incidence rate ratio between intervention and control arm
** Estimated incidence rates and incidence rate ratio between intervention and control arm adjusted for baseline score of outcome.
*** Estimated incidence rates and incidence rate ratio between intervention and control arm adjusted for baseline age, sex, socio-economic status, level of education, and score of outcome.
Incidence rate ratios below 1 indicate a protective effect of the intervention. CI = confidence interval.
