# Supplementary material for: Effects of Nurse-Led Multifactorial Care to Prevent Disability in Community-Living Older People: Cluster Randomized Trial
Source: PLoS One. 2016 Jul 26;11(7):e0158714. doi: 10.1371/journal.pone.0158714 (PMC4961429; doi:10.1371/journal.pone.0158714)
Supplement: S11 Table — (DOC) [file pone.0158714.s016.doc]

## S11 Table: Interaction terms of different levels of education, socio-economic status, baseline disability, and age

|  | **Significance level  interaction term** | **Significance level  interaction term** | **Significance level  interaction term** | **Significance level  interaction term** |
| --- | --- | --- | --- | --- |
| Level of education | intermediate p=0.94 | high p=0.39 |  |  |
| level of socio-economic status | low p=0.99 | intermediate p=0.79 |  |  |
| Baseline level of disability (Katz score) | 2-4 p=0.41 | 5-7 p=0.24 | 8-15 p=0.34 |  |
| Baseline level of age (years) | 75-79 p=0.19 | 80-84 p=0.59 | 85-89 p=0.40 | ≥90 p=0.41 |

Interaction terms for treatment × levels of education (high and intermediate), treatment × socio-economic status (low and intermediate), treatment × levels of age (quartiles), and treatment × baseline disability (tertiles).
