# Supplementary material for: Effects of Nurse-Led Multifactorial Care to Prevent Disability in Community-Living Older People: Cluster Randomized Trial
Source: PLoS One. 2016 Jul 26;11(7):e0158714. doi: 10.1371/journal.pone.0158714 (PMC4961429; doi:10.1371/journal.pone.0158714)
Supplement: S12 Table — (DOC) [file pone.0158714.s017.doc]

## S12 Table: In hours general practice care during follow-up for older persons at increased risk of functional decline

## (ISAR-PC≥2)

|  | **Intervention group**  **(%)** | **Control**  **group**  **(%)** | | **Intervention**  **group**  **mean (SD)** | **Control**  **group**  **mean (SD)** |
| --- | --- | --- | --- | --- | --- |
| **Follow-up  (6 months intervals)** | | | **GP consultation (≥1)** | | |
| 6 | 59.0 | 61.0 | | 1.2 (1.5) | 1.4 (1.8) |
| 12 | 57.1 | 55.8 | | 1.3 (1.6) | 1.3 (1.8) |
| 18 | 57.8 | 59.4 | | 1.3 (1.7) | 1.3 (1.5) |
| 24 | 56.2 | 57.5 | | 1.2 (1.6) | 1.3 (1.6) |
| **follow-up  (6 months intervals)** | **GP visit (≥1)** | | | | |
| 6 | 24.8 | 24.2 | | 0.6 (1.4) | 0.5 (1.3) |
| 12 | 24.3 | 23.2 | | 0.6 (1.7) | 0.5 (1.2) |
| 18 | 25.3 | 25.6 | | 0.5 (1.2) | 0.5 (1.2) |
| 24 | 24.5 | 22.9 | | 0.5 (1.1) | 0.5 (1.2) |

Values are numbers (percentages) unless stated otherwise. GP=general practitioner; SD=standard deviation

Values are numbers (percentages) unless stated otherwise. GP = general practitioner; SD = standard deviation.
