# Supplementary material for: Effects of Nurse-Led Multifactorial Care to Prevent Disability in Community-Living Older People: Cluster Randomized Trial
Source: PLoS One. 2016 Jul 26;11(7):e0158714. doi: 10.1371/journal.pone.0158714 (PMC4961429; doi:10.1371/journal.pone.0158714)
Supplement: S1 Text — (DOC) [file pone.0158714.s018.doc]

## S1 Text: Identification of Senior At Risk- Primary Care (ISAR-PC) screening instrument

Identification of Senior At Risk- Primary Care (ISAR-PC) is a validated screening instrument, developed to identify older community-dwelling persons (≥ 70 year) at increased risk of functional decline. The instrument is short and easy to apply. ISAR-PC consists of three questions:

| **ISAR-PC** | | |
| --- | --- | --- |
| 1. Did you need assistance on a regular basis in the last month (e.g. preparing meals, shopping, housekeeping)? | No Yes | 0.0 2.5 |
|  |  |  |
| 2 Do you regularly have memory problems? | No Yes | 0.0 2.0 |
|  |  |  |
| 3. Your age is: | 74 years or younger  Between 75 and 84 years  85 years and older | 0.0 1.5 3.0 |
| Total score | ……. | |
| Maximum score: | 7.5 points | |
| Total score 0 or 1: | Not at risk of functional decline | |
| Total score 2 or higher: | At risk of functional decline | |

The range of scores varies from 0 to 7.5 points. A score of ≥ 2 points indicates increased risk of functional decline over a one-year period. In the development cohort (n= 790) at a cut-off ≥ 2 points the AUC of the ISAR-PC ranged from 0.67 to 0.70 and the positive and negative predictive values were 48.3 and 80.5%. In the validation cohort (n= 2573) the AUC ranged from 0.63 to 0.64 and the positive and negative predictive values were 45.1 and 74.7%.[1]

Participants with a score of ≥ 2 points were eligible to participate in the FIT trial.

## References

1. Suijker JJ, Buurman BM, van Rijn M, van Dalen MT, ter Riet G, van Geloven N, et al. A simple validated questionnaire predicted functional decline in community-dwelling older persons: prospective cohort studies. Journal of clinical epidemiology. 2014;67(10):1121-30. doi: 10.1016/j.jclinepi.2014.05.014. PubMed PMID: 25103817.
