# Supplementary material for: Effects of Nurse-Led Multifactorial Care to Prevent Disability in Community-Living Older People: Cluster Randomized Trial
Source: PLoS One. 2016 Jul 26;11(7):e0158714. doi: 10.1371/journal.pone.0158714 (PMC4961429; doi:10.1371/journal.pone.0158714)
Supplement: S2 Text — (DOC) [file pone.0158714.s019.doc]

## S2 Text: Training of nurses involved in the trial

In total, 15 experienced community-care registered nurses participated in the intervention. They followed a 10-day training with specific focus on geriatric care in the community. The training was developed together with the School of Nursing from InHolland University of Applied Sciences, in collaboration with the Academic Medical Center and the Regional Council of General Practitioners, all based in Amsterdam. The training comprised of two sections of three days and one section of 4 days; 1) introduction into research and frail older persons; 2) somatic and functional geriatric conditions; and 3) psychological and social conditions. To obtain a training certificate the nurses had to fulfil a practical assignment for each section.

*Section 1:* Introduction to research focused on methodological and ethical aspects of research, participating in a randomized clinical trial, and the study protocol. Introduction to frail older persons concerned background information on ageing, care models, frailty and physical functioning, and comprehensive geriatric assessment (CGA). Subsequently, nurses were trained in conducting the CGA, drafting a care- and treatment plan (CTP) and how to formulate goals of care with patients.

*Section 2*: Somatic and functional geriatric conditions were provided by geriatric experts (e.g. general practitioners, geriatricians, pharmacist, and clinical nurse specialist) and included polypharmacy, pain management, malnutrition, fall prevention, incontinence, sleep disorder, and ADL and IADL impairments.

*Section 3:* The training in psychological and social conditions was also provided by content experts and consisted of caregiver burden, cognitive impairment, depression, financial problems, and elder abuse.

Across the three modules the training focused on how to apply evidence-based interventions, how to provide patient-centred care and empower the older person.

Every six weeks, the nurses attended a refresher course on adherence to the study protocol, conducting the CGA, making a care- and treatment plan, formulating goals of care with patients, patient-centred care and empowerment of the older person. Two additional afternoons were spent on communication training provided by an expert, applying principles of motivational interviewing and complex situations (e.g. the informal caregiver has other wishes than the patient).
