# Supplementary material for: Effects of Nurse-Led Multifactorial Care to Prevent Disability in Community-Living Older People: Cluster Randomized Trial
Source: PLoS One. 2016 Jul 26;11(7):e0158714. doi: 10.1371/journal.pone.0158714 (PMC4961429; doi:10.1371/journal.pone.0158714)
Supplement: S3 Text — (DOC) [file pone.0158714.s020.doc]

## S3 Text: Evidence-based protocols used in the trial

To create uniformity in diagnostic assessments and interventions, a multidisciplinary expert panel developed a toolkit that underpins the individually tailored care and treatment plan (CTP). The toolkit was constructed based on previous experience from the DEFENCE study. The toolkit consisted of standardized protocols for the 24 most prevalent geriatric conditions in the CGA following international guidelines and were based on evidence or on current best practice.[2] The protocols are available on a website: <http://www.effectieveouderenzorg.nl/Toolkit.aspx> [in Dutch] The nurses could apply the evidence-based protocols after the initial CGA. If the older person recognized the geriatric conditions, that resulted from the CGA, and identified these conditions as a priority, the evidence-based protocols were subsequently used.

The protocols were a practical translation of existing guidelines. They consisted of further diagnostic assessments, a stepwise overview of risk factors, and evidence-based interventions that the older person him/herself, the nurse, the GP or other healthcare professionals could perform. For example, if a person had previously fallen, risk factors for falling, such as visual impairment or medication side-effects, were assessed, and the nurses were guided to make an overview to address the most important diagnostic or prognostic factors. Subsequently, they were guided to propose interventions to the GP or the geriatrician such as physiotherapy, evaluation of visual impairment or evaluation of medication. Furthermore, for each geriatric condition also background and additional in-depth information was available, such as prevalence, risk factors, screening, diagnostic assessment and interventions.

## References

2. Buurman BM, Hoogerduijn JG, de Haan RJ, Abu-Hanna A, Lagaay AM, Verhaar HJ, et al. Geriatric conditions in acutely hospitalized older patients: prevalence and one-year survival and functional decline. PloS one. 2011;6(11):e26951. doi: 10.1371/journal.pone.0026951. PubMed PMID: 22110598; PubMed Central PMCID: PMC3215703.
