# Supplementary material for: Effects of Nurse-Led Multifactorial Care to Prevent Disability in Community-Living Older People: Cluster Randomized Trial
Source: PLoS One. 2016 Jul 26;11(7):e0158714. doi: 10.1371/journal.pone.0158714 (PMC4961429; doi:10.1371/journal.pone.0158714)
Supplement: S4 Text — (DOC) [file pone.0158714.s021.doc]

## S4 Text Care as usual in the Dutch healthcare system

In the Dutch healthcare system, the general practitioner (GP) plays a central role; as the gatekeeper of the healthcare system, s/he is the first and only freely accessible medical professional, and people are used to visiting their GP first if they have a health problem.[3] In the last two decades, there has been an increasing task delegation from GP to registered nurses working in GP practice. Evidence-based protocols for care provided by GPs are available for the management of chronic conditions such as diabetes mellitus, cardiovascular disease, COPD, and obesity. In 2001, the average adherence rate to these protocols was 67% with a large variation.[4] In 2010, the healthcare insurer started to reimburse preventive primary care for community-dwelling frail older people and GPs began to employ qualified nurses specialized in providing such preventive care. In the control group, we offered practices a temporary reimbursement to postpone this nurse-led care for older people until study termination. Throughout the study, we monitored all participants’ healthcare and home care utilization (Table1).

## References

3. Jones R, Schellevis F, Westert G. The changing face of primary care: the second Dutch national survey. Family practice. 2004;21(6):597-8. Epub 2004/10/07. doi: 10.1093/fampra/cmh603. PubMed PMID: 15465880.

4. Grol R. Successes and failures in the implementation of evidence-based guidelines for clinical practice. Medical care. 2001;39(8 Suppl 2):Ii46-54. Epub 2001/10/05. PubMed PMID: 11583121.
